# High-power pulsed-laser ablation of aluminum alloy: Simulation model and experimental validation

**DOI:** 10.1371/journal.pone.0357262

**Published:** 2026-09-03

**Authors:** Qimeng Chen, Boxiang Hou, Yan Li, Zhe Wang, Jian Zhao, Keyan Dong

**Affiliations:** 1 Changchun University of Science and Technology The School of Electro-Optical Engineering, Changchun, Jilin, China; 2 Changchun Jingyi Optoelectronics Technology Company, Changchun, Jilin, China; University of Sharjah, UNITED ARAB EMIRATES

## Abstract

To address the critical influence of pulse shape on the laser ablation of metallic materials, this study proposes a comprehensive methodology that integrates finite‑element numerical simulations with experimental validation. Based on the spatiotemporal energy‑distribution characteristics of Gaussian and super‑Gaussian pulses, the temperature evolution, plasma electron density, and ablation efficiency of 6061 aluminum alloy were systematically analyzed under various single‑pulse energies and spot radii. Comparison between experimental measurements and numerical predictions enables the construction of an energy‑coupling model for laser ablation and establishes a theoretical framework describing how pulse shape governs the material’s thermal response and plasma dynamics. The performance of the experimental laser was benchmarked against that of a commercial laser. The results show that the maximum difference in dispersion coefficients between the two lasers is only 0.19%, and the largest discrepancy in average perforation time between the Gaussian and super‑Gaussian pulses is approximately 6 s. Owing to its more uniform energy distribution, the super‑Gaussian pulse exhibits 3–18% higher energy‑coupling efficiency and material‑removal efficiency under identical energy input. Therefore, controlling the pulse shape can significantly enhance the stability and efficiency of laser ablation, satisfy the requirements of high‑precision material processing regarding energy‑distribution uniformity and ablation consistency, and provide a theoretical foundation for applying pulse‑shaping techniques in high‑efficiency laser manufacturing.

## Introduction

Laser ablation, as an efficient and controllable technique for material removal and surface modification, has been widely applied in aerospace engineering, precision manufacturing, and structural fabrication [1]. During the interaction between a pulsed laser and metallic materials, the energy-deposition rate, heat-conduction behavior, and plasma generation and evolution are strongly coupled, rendering the ablation response highly sensitive to laser parameters [2]. In particular, the mechanism by which the pulse-shape profile affects the material’s thermal response and plasma dynamics remains insufficiently understood and lacks systematic investigation.

Laser ablation, as an efficient and controllable technique for material removal and surface modification, has been widely applied in aerospace engineering, precision manufacturing, and structural fabrication [1]. During the interaction between a pulsed laser and metallic materials, the energy‑deposition rate, heat‑conduction behavior, and plasma generation and evolution are strongly coupled, rendering the ablation response highly sensitive to laser parameters [2]. In particular, the mechanism by which the temporal pulse‑shape profile affects the material’s thermal response and plasma dynamics remains insufficiently understood and lacks systematic investigation.

Existing studies primarily focus on the influence of parameters such as single-pulse energy, pulse width, and spot size on ablation characteristics, whereas the intrinsic role of pulse shape has received comparatively limited attention. In 2021, Naveed Ullah et al. [3] developed an ultraviolet nanosecond laser micromachining model for polycrystalline diamond (PCD) materials, establishing a linear relationship between pulse-energy density and ablation-pit diameter while incorporating the Gaussian intensity distribution of the beam cross-section. Their results indicated that the ablation-threshold energy density of PCD is approximately 3.8-4.2J/cm², with a goodness of fit of R² ≈ 0.97 between crater diameter and energy density. In 2023, A. Andrásik et al. [4] investigated the dependence of the ablation-threshold fluence of BK7 optical glass on spot radius, elucidated the variation of optical-response modes with pulse energy, and identified plasma-mirror formation as the dominant factor governing the delamination threshold. Their study reported that the ablation threshold of BK7 is approximately 2.1-2.5J/cm² when the spot radius is below 10μm. As the radius increases to 30μm, the threshold decreases to 0.6–0.8J/cm², demonstrating a pronounced size effect. In 2025, Shunping Li et al. [5] reported that the extent of ablation and melting during nanosecond near-infrared laser–silicon interaction is governed by the pulse-energy deposition rate. They found that for pulse widths of 10–20 ns, the peak power reaches 10 kW and the surface-temperature rise exceeds 2000K, thereby promoting ablation. When the pulse width is extended to 100–200 ns, the peak power decreases by a factor of 5–10, and the surface temperature stabilizes at 1400-1600K, favoring melting rather than ablation. Consequently, short-pulse irradiation is more likely to induce surface ablation, whereas long-pulse irradiation tends to promote melting.

Although numerous studies have examined the influence of laser parameters on ablation behavior, systematic and quantitative analyses of how pulse-shape variations affect metal ablation remain scarce. Gaussian pulses are widely employed due to their natural output profile; however, their spatial energy distribution is strongly center-peaked, often leading to excessively localized energy deposition [6]. In contrast, super-Gaussian pulses exhibit a flat-top energy distribution that provides a more spatially uniform irradiation profile, thereby potentially reducing the heat-affected zone and enhancing energy-utilization efficiency [7]. However, comparative studies evaluating the two pulse shapes in terms of ablation depth, plasma-electron-density evolution, and perforation efficiency remain insufficient.

Although the above studies have provided a foundation for understanding laser‑ablation mechanisms, quantitative descriptions and experimental validations of pulse‑shape effects remain incomplete. Motivated by these gaps, this work investigates 6061 aluminum alloy and develops a two‑dimensional, axisymmetric, transient multiphysics coupling model of pulsed‑laser irradiation using finite‑element analysis. The temperature‑field evolution and plasma electron‑density dynamics induced by Gaussian and super‑Gaussian pulses are systematically analyzed under varying single‑pulse energies and spot radii. In parallel, controlled experiments measuring ablation depth and perforation time are conducted to validate the accuracy and applicability of the proposed model. The objective of this study is to elucidate the key mechanisms by which pulse shape influences the laser‑ablation process and to provide both theoretical insights and experimental evidence for optimizing precision‑machining parameters of lightweight metallic materials.

## Theory and model

### Basic principle

**Fig 1:**
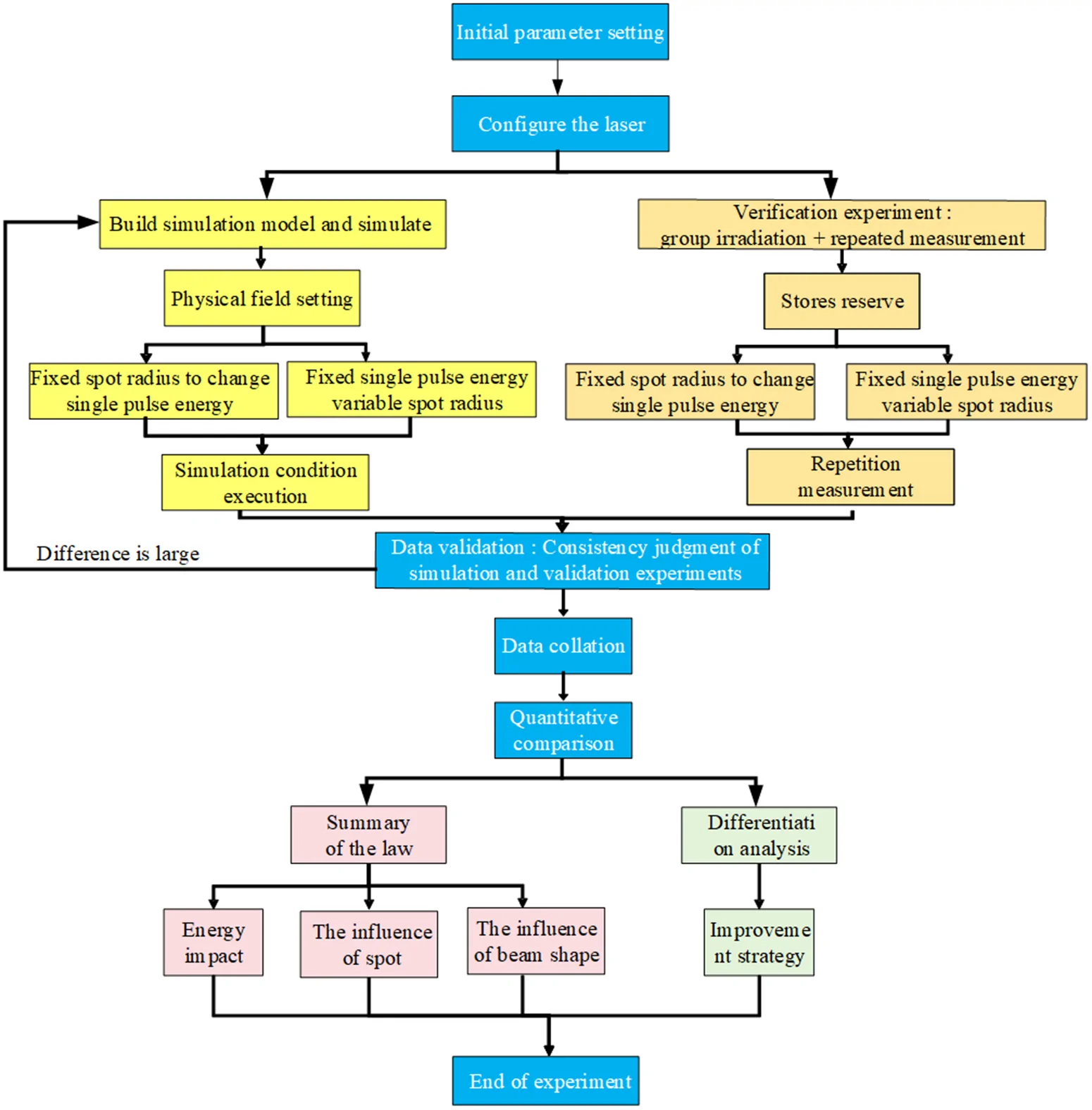
Technology roadmap.

In laser ablation, the pulse shape of a pulsed laser exerts a far more pronounced influence on the processing outcome than continuous-wave laser irradiation. Common pulse shapes include Gaussian, super-Gaussian, and custom-shaped waveforms [8]. Variations in pulse shape fundamentally alter key processing parameters—such as energy-deposition rate, thermal-diffusion depth, and phase-transition behavior—and consequently influence processing efficiency, surface quality, and the extent of the heat-affected zone [9]. To systematically examine the influence of these parameters and optimize the ablation process, a detailed technical workflow was developed, with its core steps illustrated in Fig 1. The workflow encompasses numerical-simulation modeling, controlled-variable experimentation, experimental validation, and final synthesis of governing principles and optimization strategies.

### Effect of pulse shape on energy deposition

To systematically investigate the influence of pulse shape on the laser-ablation process, we quantitatively analyze the evolution of energy-deposition characteristics and thermal-diffusion behavior under different temporal pulse profiles using the following theoretical framework. The relationship between the time-integrated laser pulse energy as:


$$\begin{array}{c} Q=\int_{\text{0}}^{\tau }P(t)dt \end{array}$$ (1)

where P(t) is the time-dependent laser power, τ is the pulse duration [10]. Variations in pulse shape lead to substantial changes in the energy‑deposition rate, which in turn strongly influence the thermal response of the material. This thermal response can be described by the thermal‑diffusion equation:


$$\begin{array}{c} \rho C_{p}\frac{\partial T}{\partial t}=\nabla \cdot (k\nabla T)+S(r,z,t) \end{array}$$ (2)

where T (unit: K) denotes temperature, k is the thermal conductivity, ρ is the material density, $C_{p}$ is the specific heat capacity, and S(r,z,t) represents the volumetric heat source [11]. A two-dimensional axisymmetric cylindrical coordinate system (r,z) is adopted, where r is the radial coordinate and z is the depth measured from the initial target surface (z = 0). Differences in temporal energy distribution among pulse shapes significantly influence melt-pool formation kinetics and the resulting material-removal mechanisms. These thermophysical processes are further elucidated through numerical simulations.

### Effect of pulse shape on plasma dynamics

To systematically investigate the influence of pulse shape on plasma behavior during laser ablation, we quantitatively analyze plasma initiation, evolution, and laser–plasma interactions under different temporal pulse profiles using the following theoretical framework. The plasma‑initiation threshold and subsequent shielding effects are strongly governed by the temporal distribution of pulse power density. The critical electron density for plasma formation can be expressed as:


$$\begin{array}{c} n_{c}=\frac{\epsilon_{\text{0}}m_{e}\omega^{\text{2}}}{e^{\text{2}}} \end{array}$$ (3)

where $\epsilon_{\text{0}}$ is the vacuum permittivity, $m_{e}$ is the electron mass, ω is the laser angular frequency, and e is the electron charge. When the laser power density exceeds a critical threshold, the ablated material undergoes rapid ionization, forming a plasma [12]. The absorption and reflection of subsequent incident laser energy by this plasma play a decisive role in determining processing efficiency. The absorption coefficient can be expressed as:


$$\begin{array}{c} \alpha_{pl}\propto \frac{\nu_{ei}}{\omega^{\text{2}}+\nu_{ei}^{\text{2}}}n_{e} \end{array}$$ (4)

where $\nu_{ei}$ is the electron–ion collision frequency and $n_{e}$ is the electron density. The pulse shape directly governs the onset, evolution of electron density, and lifetime of the plasma by modulating the rate of energy injection [13]. Pulses with steep leading edges reach the plasma-initiation threshold more rapidly, whereas flat-top pulses sustain a more stable plasma plume for a longer duration. It should be emphasized that the plasma‑related equations are not fully coupled into the finite‑element solver; they are employed to interpret plasma generation and shielding trends based on the simulated thermal field rather than to resolve the plasma plume dynamics.

## Numerical calculation

### Numerical calculation model

In this work, the finite‑element method is employed to solve the transient heat‑transfer equation with phase change in a two‑dimensional axisymmetric domain. The governing equation is the energy conservation equation, where the temperature‑dependent thermophysical properties of the aluminum alloy are incorporated. Phase transition from solid to liquid is modeled using an effective heat‑capacity (enthalpy) method. Material removal is identified when the local temperature exceeds the boiling temperature, and the corresponding elements are excluded from subsequent thermal conduction

A numerical model of pulsed-laser irradiation on 6061 aluminum alloy was established using finite-element analysis. The dimensions of the aluminum-alloy specimen were set to L = 50 mm and W = 30 mm. The pulsed-laser parameters used in the numerical simulation included a repetition frequency of f = 100 Hz; single pulse energy E_1_ = 50 mJ, E_2_ = 100 mJ, E_3_ = 150 mJ; the spot radius R_1_ = 1 mm, R_2_ = 1.5 mm, R_3_ = 2 mm. The laser wavelength is 1064 nm, and the pulse duration is 10 ns. The super-Gaussian pulse is defined as $I(r)=I_{\text{0}}exp[-{\left(r/R\right)}^{n}]\;$where n = 8 denotes the super-Gaussian order. The pulsed laser was assumed to be normally incident at the center of the aluminum-alloy surface. Upon irradiation, the absorbed laser energy is converted into heat and subsequently transported within the material, leading to a transient rise in both surface and subsurface temperature.

The finite‑element model was built using 2D axisymmetric triangular elements with quadratic Lagrange shape functions. The mesh consisted of approximately 15,000 elements after refinement; the minimum element size in the laser‑irradiated region was 5 μm. A mesh‑independence study was performed by comparing results with 8,000‑element and 25,000‑element meshes: the maximum temperature variation was less than 2%, confirming that the 15,000‑element mesh provided an adequate balance between accuracy and computational cost.

Boundary conditions were defined as follows:

On the symmetry axis (𝑟 = 0: thermal insulation (zero radial heat flux).On the top surface: combined convective heat loss with a coefficient h = 10W ⋅ m^-2^ ⋅ K^-1^and surface‑to‑ambient radiation with emissivity *ε* = 0.2; the ambient temperature was set to 293 K.On the bottom and outer side boundaries: fixed temperature T = 293 K.

The time‑dependent solver employed adaptive time stepping, with an initial step of 1 × 10^−2^ s, to capture the rapid thermal transients during the nanosecond pulse. The nonlinear system was handled using a Newton–Raphson iterative scheme with a relative tolerance of 1 × 10^−5^. The enthalpy method was used for solid–liquid phase change, and elements whose temperature exceeded the boiling point were deactivated to simulate material removal.

The finite-element approach adopted in this study is suitable for resolving the transient thermal response and phase-transition behavior under nanosecond pulsed-laser irradiation. However, it does not explicitly account for molten-material flow, recoil pressure, or plasma plume hydrodynamics. These effects are implicitly reflected through effective energy deposition and post-processed plasma-density estimates.

### Numerical calculation parameters

**Fig 2:**
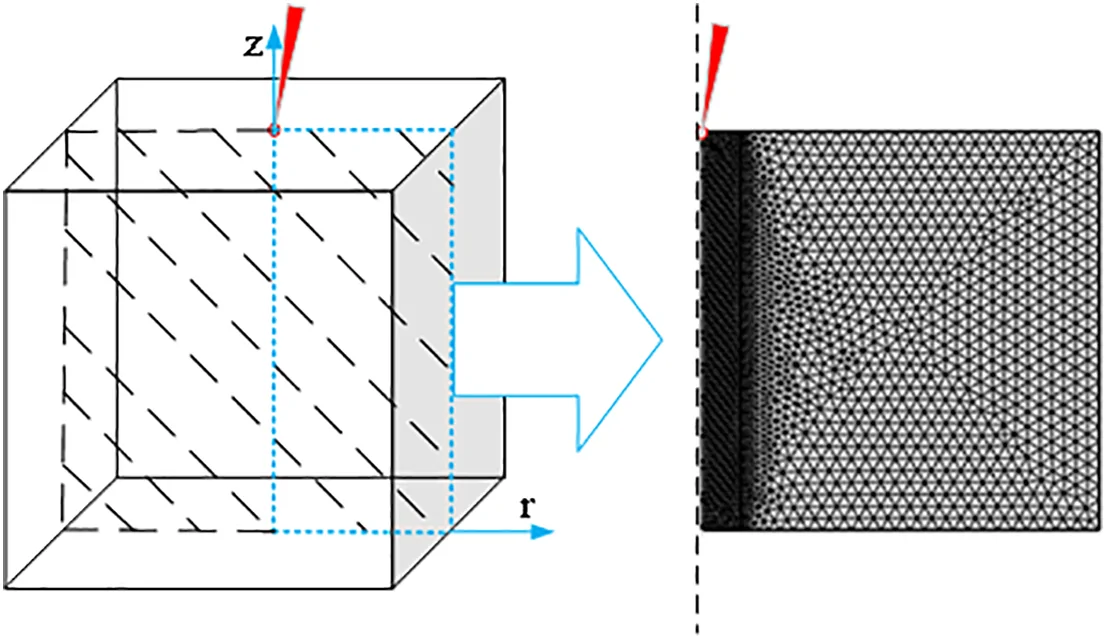
Geometric modeling diagram of ablative aluminum alloy.

The model was solved using finite-element simulation. During meshing, the grid density was refined near the heat source to ensure adequate spatial resolution. For the two-dimensional model, a triangular mesh was applied to discretize both the symmetry plane of the irradiated region and the central area of the target. The geometric model is shown in Fig 2.

The thermophysical properties of the aluminum alloy used in the numerical simulation are listed in Table 1. The thermophysical properties listed in Table 1 are incorporated into the finite-element model as temperature-dependent parameters, allowing continuous transition between solid and liquid states through the enthalpy method.

**Table 1:** Physical property parameters of aluminum alloy.

Physical Behavior	Numerical Value	Physical Behavior	Numerical Value
Solid density(kg/m^3^)	2700	liquid density(kg/m^3^)	2385
Solid thermal conductivity(m·K)	160	Liquid thermal conductivity(m·K)	100
Solid specific heat capacity(kg·K)	917	Liquid specific heat capacity(kg·K)	1080
Melting latent heat(J/kg)	3.896 × 10^5^	latent heat of vaporization(J/kg)	9.462 × 10^6^
Melting point(K)	933	boiling point(K)	2793
dynamic viscosity(Pa·s)	1.2 × 10^−3^	Radiative emissivity	0.2

The absorptivity of the aluminum alloy increases with temperature. According to previously reported data and literature [14], the temperature-dependent absorptivity of 6061 aluminum alloy can be approximated as:


$$\begin{array}{c} A(T)=\left\{ \begin{array}{c} \text{0}.\text{354}\sqrt{-\text{1}.\text{0}+\text{0}.\text{0125}T}\;\;\;\;\;\;\;\text{293}\leq T<\text{908}\\ \text{0}.\text{354}\sqrt{\text{10}.\text{7}+\text{0}.\text{0145}T}\;\;\;\;\;\;\text{908}\leq T<\text{2793} \end{array}\right. \end{array}$$ (5)

In addition, to provide a more complete material description, the chemical composition and basic mechanical properties of 6061 aluminum alloy are summarized in Table 2.

**Table 2:** Chemical composition and mechanical properties of 6061 aluminum alloy.

Element/ Property	Value
Si (wt%)	0.4–0.8
Fe (wt%)	≤0.7
Cu (wt%)	0.15–0.4
Mn (wt%)	≤0.15
Mg (wt%)	0.8–1.2
Cr (wt%)	0.04–0.35
Zn (wt%)	≤0.25
Ti (wt%)	≤0.15
Al (wt%)	Balance
Tensile strength (MPa)	310 (min)
Yield strength (MPa)	276 (min)
Elongation (%)	12–17
Hardness (HB)	95

**Fig 3:**
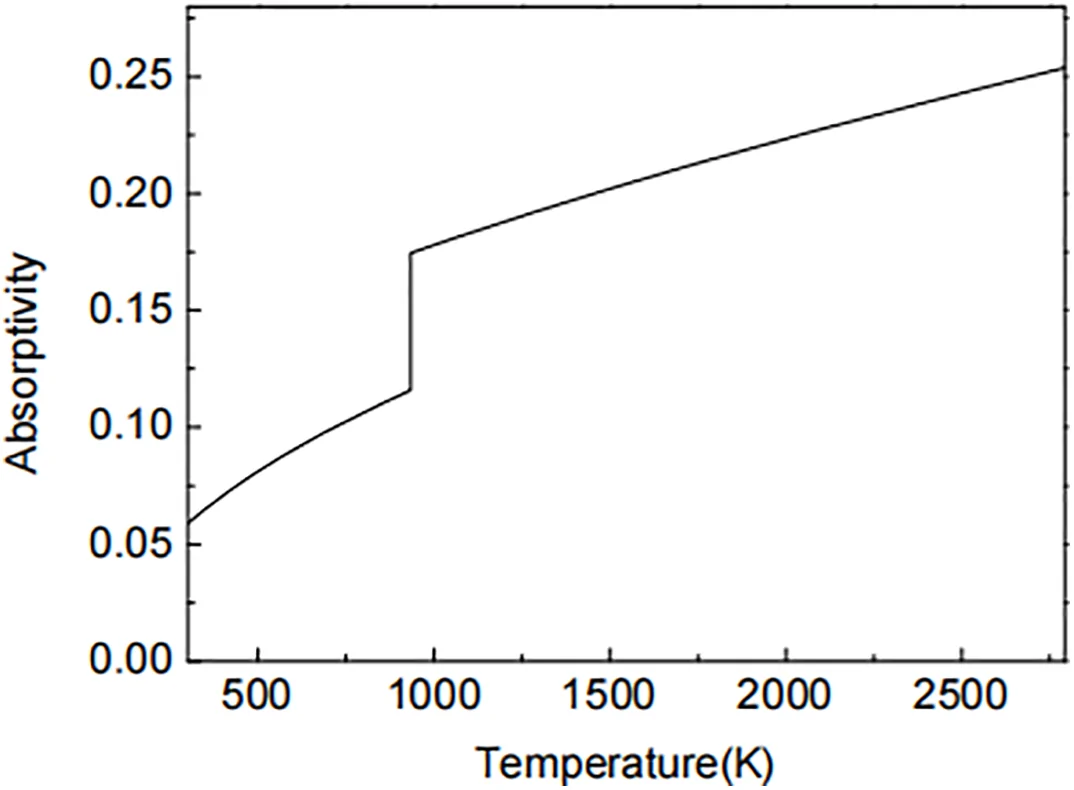
Relationship between surface absorptivity of aluminum alloy and temperature.

The curve of the absorption rate of aluminum alloy with temperature is shown in the Fig 3:

### Simulation experiment of the influence of pulse shape on ablative aluminum alloy

To systematically evaluate the influence of different pulse shapes on the laser-ablation process, the ablation morphology of 6061 aluminum alloy was compared with multiphysics numerical-simulation results under varying single-pulse energies and spot radii. The simulations considered two representative pulse shapes: Gaussian and super-Gaussian. Fixed-point ablation on the aluminum-alloy surface was examined under different pulse shapes, single-pulse energies, and spot radii.

#### Relationship between pulse shape and ablation depth.

To investigate how different laser-beam types influence the ablation behavior and thermal response of aluminum-alloy materials, this section presents a series of controlled-variable simulation experiments. Gaussian and super-Gaussian beams were used as the laser heat sources. With all other laser parameters held constant, the single-pulse energy and spot radius were varied as the primary independent variables. For each beam type and each combination of single-pulse energy and spot radius, the laser irradiated the aluminum-alloy surface in a single-pulse mode. The primary observables were the ablation morphology and the transient temperature evolution of the aluminum alloy during and after laser irradiation.

**Fig 4:**
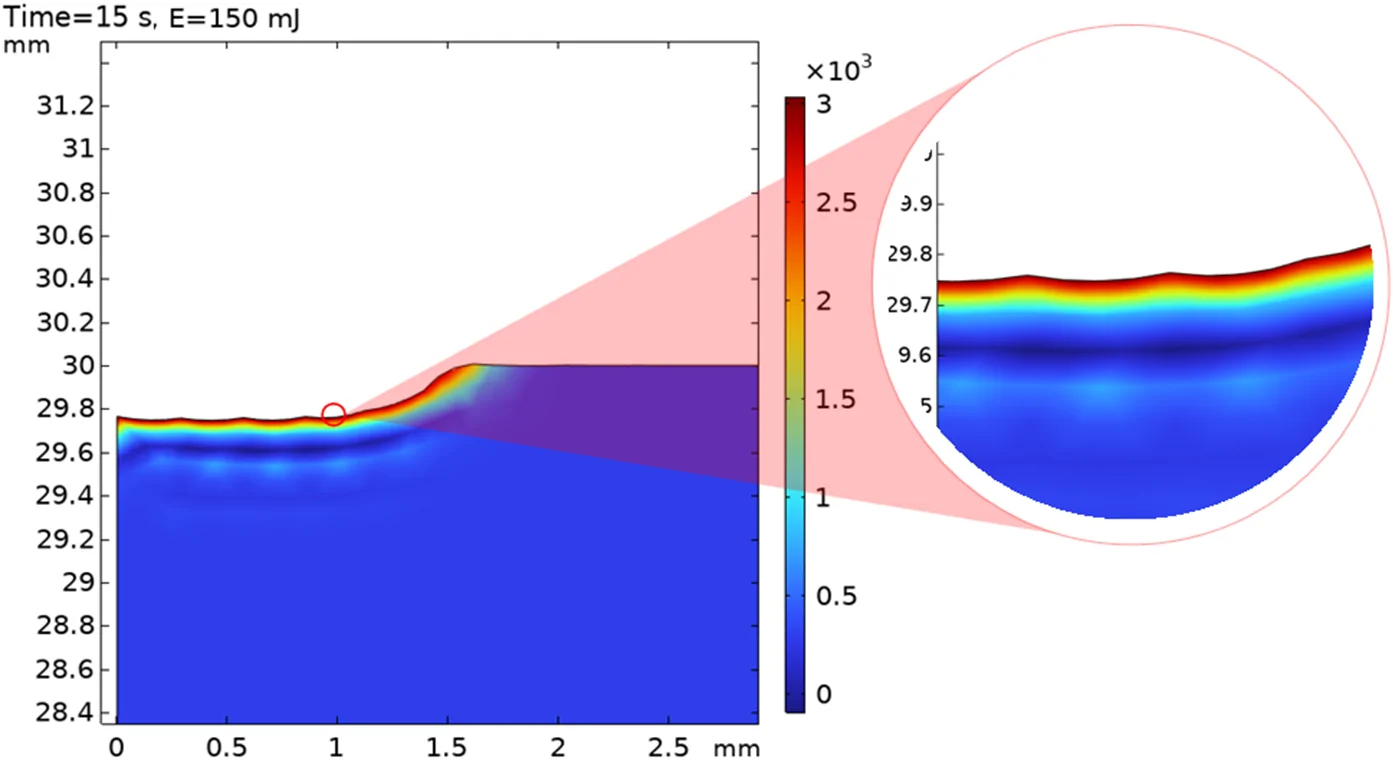
Laser ablation results and details.

**Fig 5:**
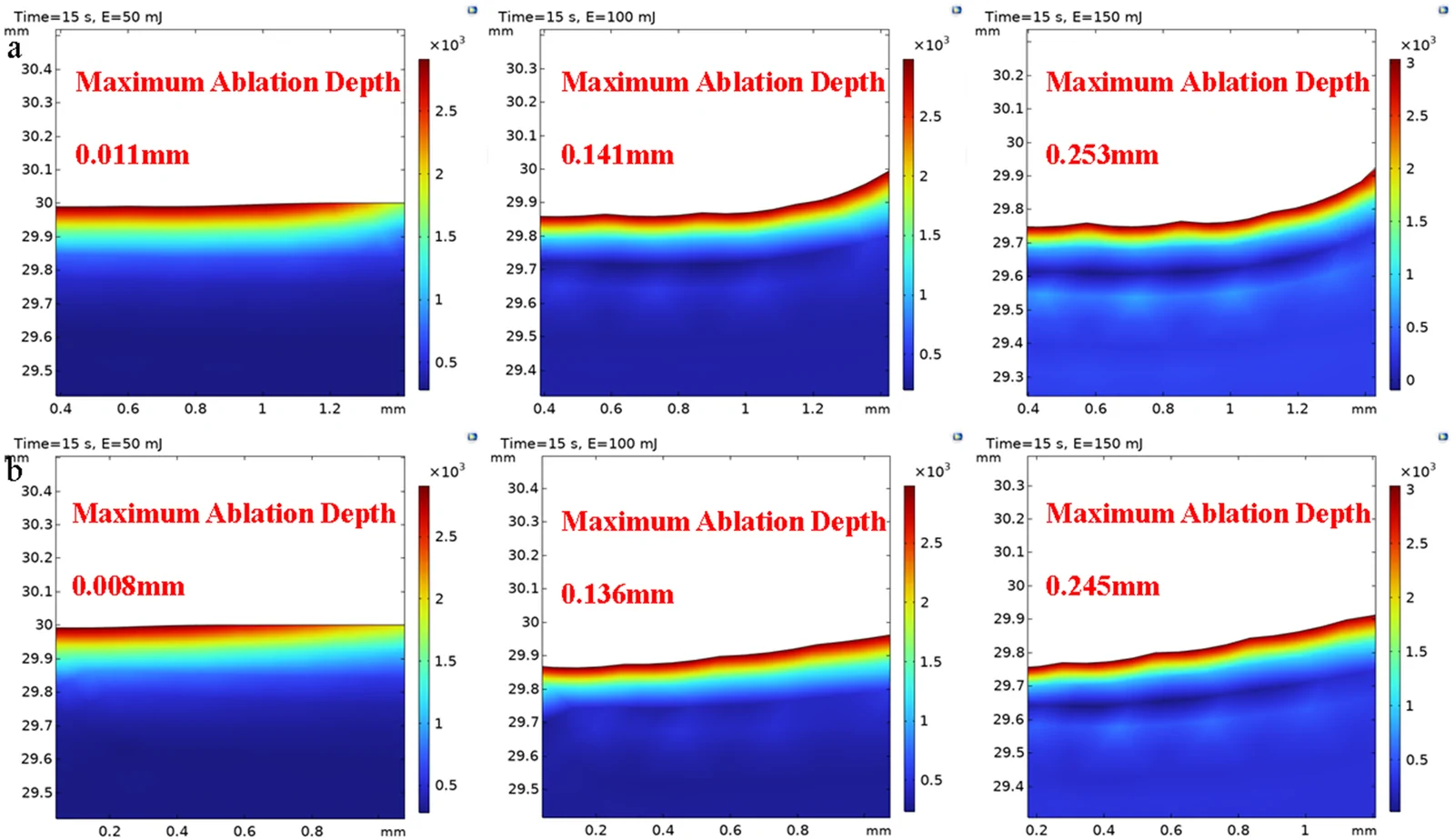
The ablation depth of laser irradiated aluminum alloy under different single pulse energy (upper super Gaussian, lower Gaussian).

Ablation depth serves as a key indicator for evaluating the effectiveness of laser processing. The initial flat surface of the aluminum alloy target is defined as the zero reference plane (z = 0), and the ablation depth is evaluated as the maximum downward displacement of the material surface relative to this plane.The ablation morphology observed at the end of irradiation is presented in Figs 4 and 5. These figures illustrate representative cross-sectional profiles of the ablation pits formed under each condition and highlight key features, including the ablation-pit depth produced by different beam types. Quantitative analysis of these morphologies particularly the pit depth provides critical insight into how beam-energy distribution governs the material-removal mechanism and informs optimization of laser-processing parameters.

**Fig 6:**
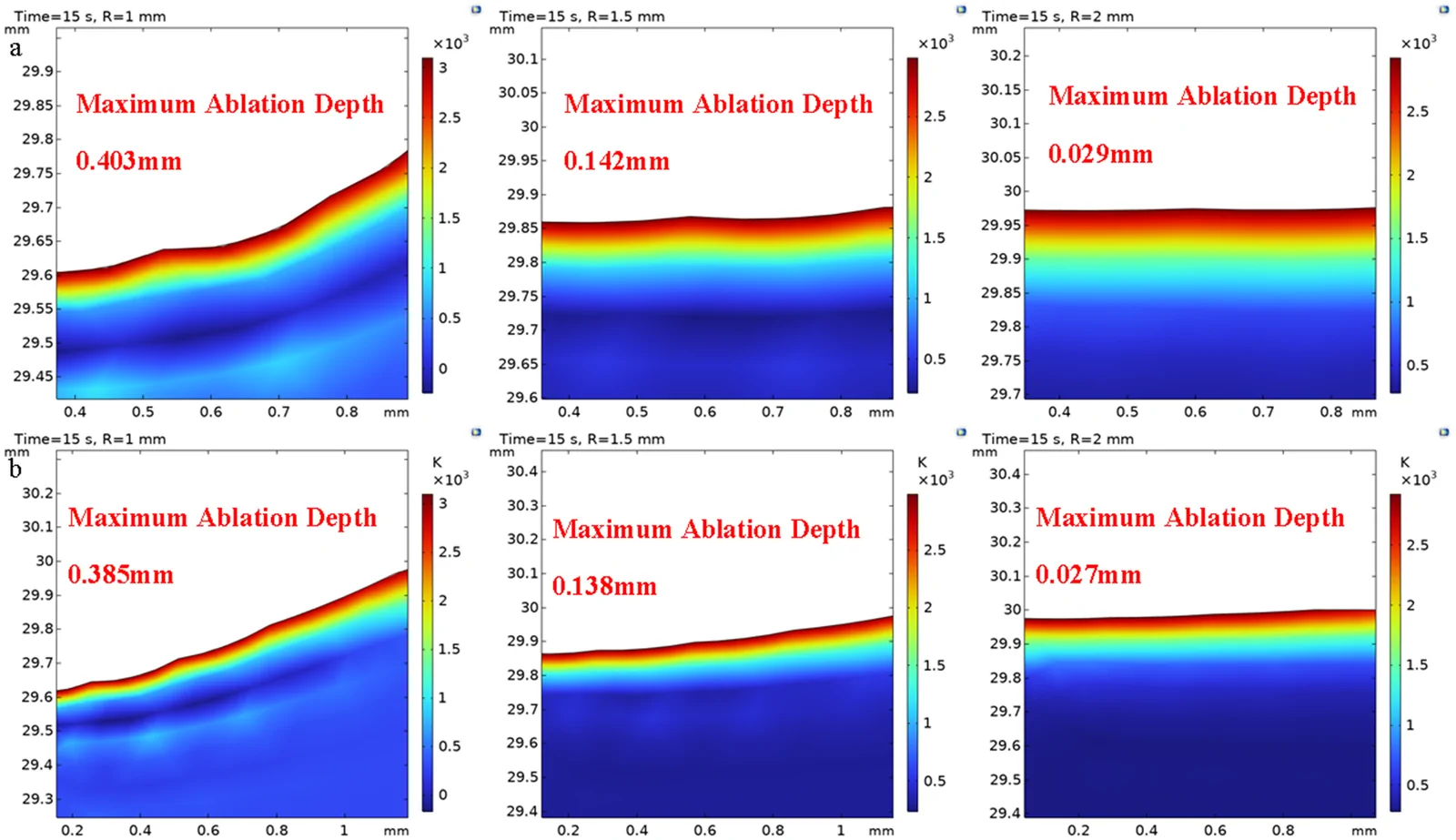
The ablation depth of laser irradiated aluminum alloy under different spot radii (upper super Gaussian, lower Gaussian).

In Fig 4, the critical regions of the laser ablation profile are locally magnified, emphasizing the maximum ablation depth achieved under the corresponding conditions. This visualization provides a more intuitive comparison of the depth variations at the bottom of the ablation crater. Figs 5 and 6 further present enlarged views of the maximum ablation depth obtained under different experimental parameters. By examining the locally enlarged cross-sectional morphologies across various conditions, the evolution of ablation depth with changing parameters can be clearly identified and quantitatively compared.

The simulation results indicate that, for a given beam type, the ablation depth of aluminum alloy increases significantly as the single-pulse energy rises from 50 to 150 mJ. At identical single-pulse energies, the ablation depth produced by the super-Gaussian beam consistently exceeds that of the Gaussian beam, with the advantage being most pronounced at lower energy levels. For both beam types, the ablation depth decreases markedly as the spot radius increases from 1 to 2 mm. At a fixed spot radius, the super Gaussian beam consistently produces a greater ablation depth than the Gaussian beam.

These results confirm that the flat-top spatial-energy distribution of the super-Gaussian beam yields higher material-removal efficiency than the Gaussian beam under equivalent energy input. This advantage becomes particularly evident when the energy is near the ablation threshold, where improved coupling efficiency plays a dominant role.

#### Relationship between pulse shape and plasma electron density.

**Fig 7:**
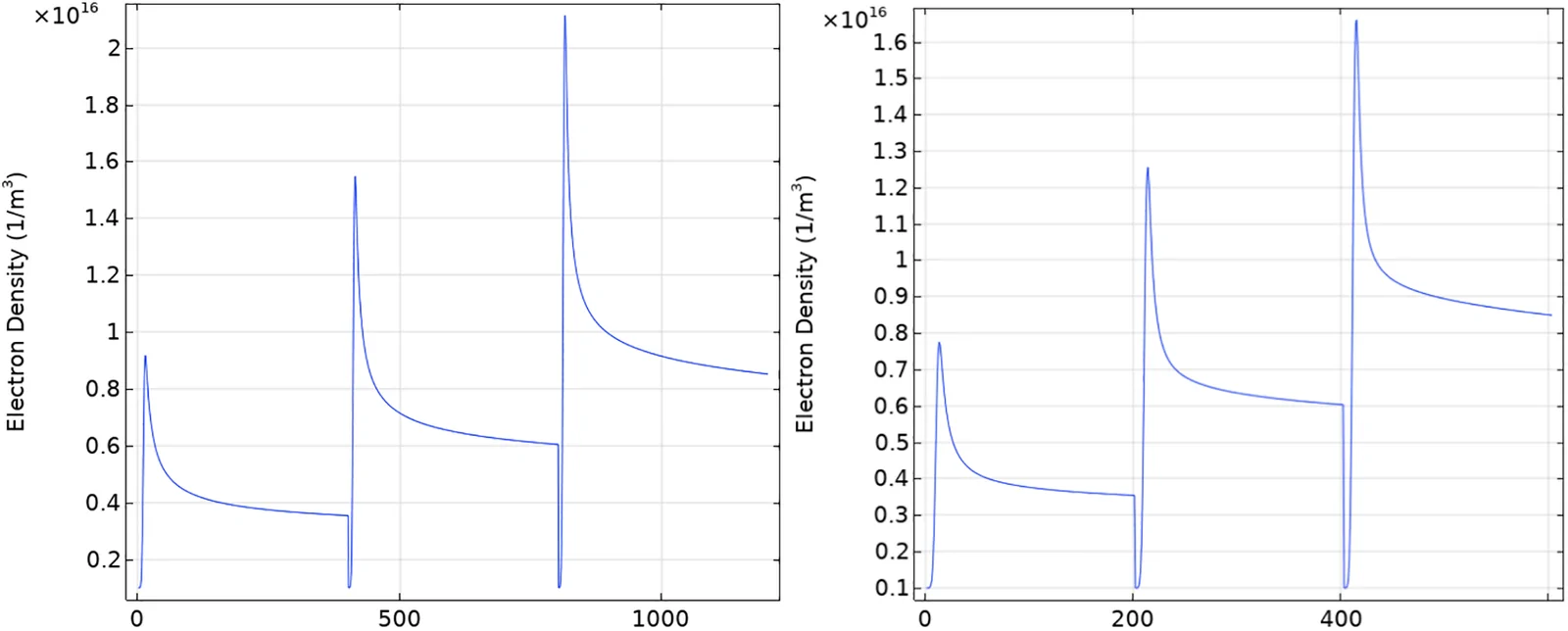
Electron density of plasma induced by different single pulse energy (Gaussian pulse on the left, super Gaussian pulse on the right).

Figs 6 and 7 present the temporal evolution of plasma electron density for different pulse shapes, single-pulse energies, and spot radii. The electron-density evolution provides insight into plasma-induced attenuation of the laser and supports optimization of laser-processing parameters.

The evolution of electron density is governed by a convection–diffusion–reaction equation of the form:


$$\begin{array}{c} \frac{\partial n_{e}}{\partial t}+\nabla \cdot {\vec{\Gamma }}_{e}=R_{e} \end{array}$$ (6)

The first term $\frac{\partial n_{e}}{\partial t}\;$in the transport equation describes the rate of change of the electron density at a given point in space. This transient term is essential for capturing the dynamics of nanosecond- to femtosecond-scale laser pulses. The second term $\nabla \cdot {\vec{\Gamma }}_{e}\;$represents the spatial transport (convection and diffusion) of electrons, i.e., how electrons move from one location to another.

The Poisson equation ensures that, when local charge neutrality in the plasma is broken, a self-consistent electric field is established. This bipolar field is critical to the expansion dynamics of the plasma.


$$\begin{array}{c} -\nabla \cdot (\epsilon_{\text{0}}\nabla V)=\rho =e(n_{i}-n_{e}) \end{array}$$ (7)

In laser-induced plasmas, steep initial electron–ion density gradients drive rapid outward diffusion of electrons due to their low mass and high mobility. This results in an ion-rich region at the plasma boundary and an inward-directed electric field. The resulting electric field accelerates ion motion while constraining excessive electron diffusion, thereby maintaining quasi-neutrality within the plasma.

**Fig 8:**
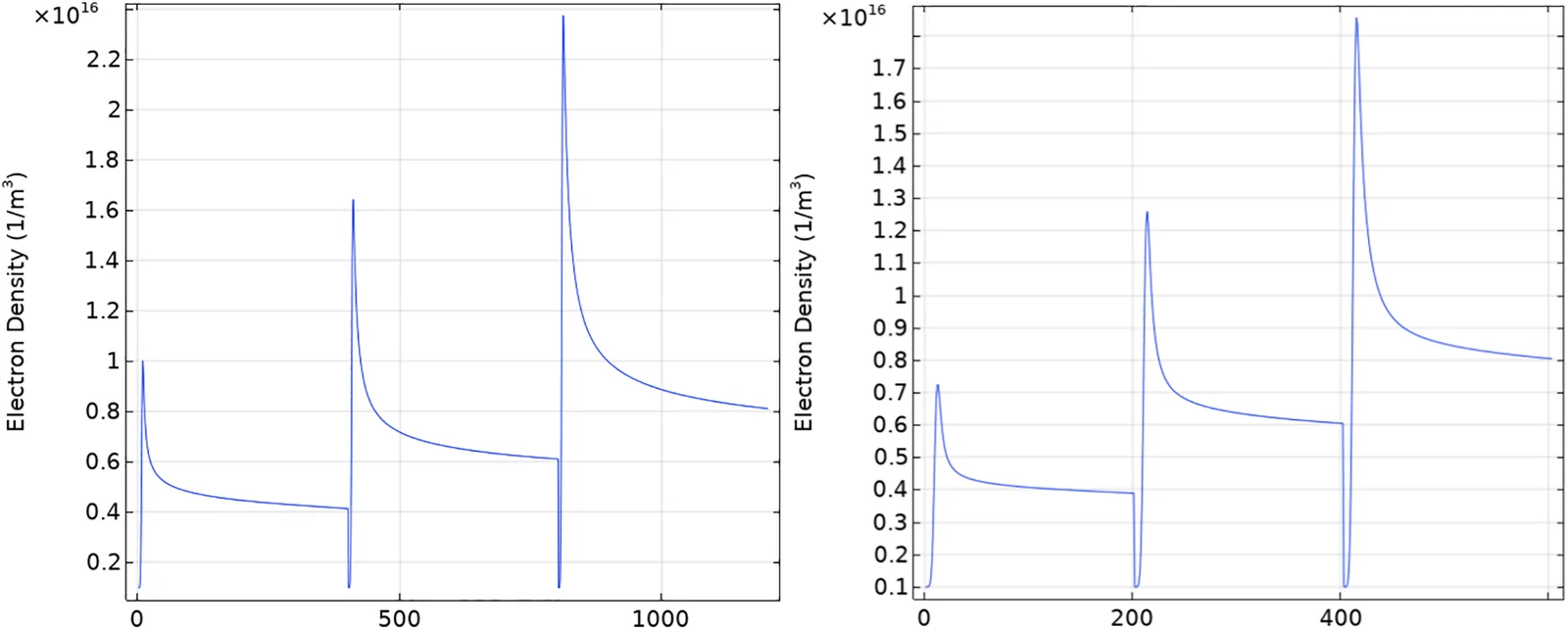
Plasma electron density induced by different spot radii (Gaussian pulse on the left, super-Gaussian pulse on the right).

Figs 7 and 8 compare the plasma electron density under varying single-pulse energies and spot radii. The single-pulse energies investigated were 50, 100, and 150 mJ. The corresponding spot radii were 1, 1.5, and 2 mm.

The results of plasma electron density are summarized as shown in Tables 3 and 4:

**Table 3:** Electron density under different single pulse energy irradiation.

	50 mJ	100 mJ	150 mJ
Gaussian	9.10e15	1.55e16	2.11e16
Super Gaussian	7.80e15	1.25e16	1.65e16

**Table 4:** The results of plasma density under different spot radius.

	1 mm	1.5 mm	2 mm
Gaussian	1e16	1.64e16	2.37e16
Super Gaussian	7.24e15	1.26e16	1.86e16

**Fig 9:**
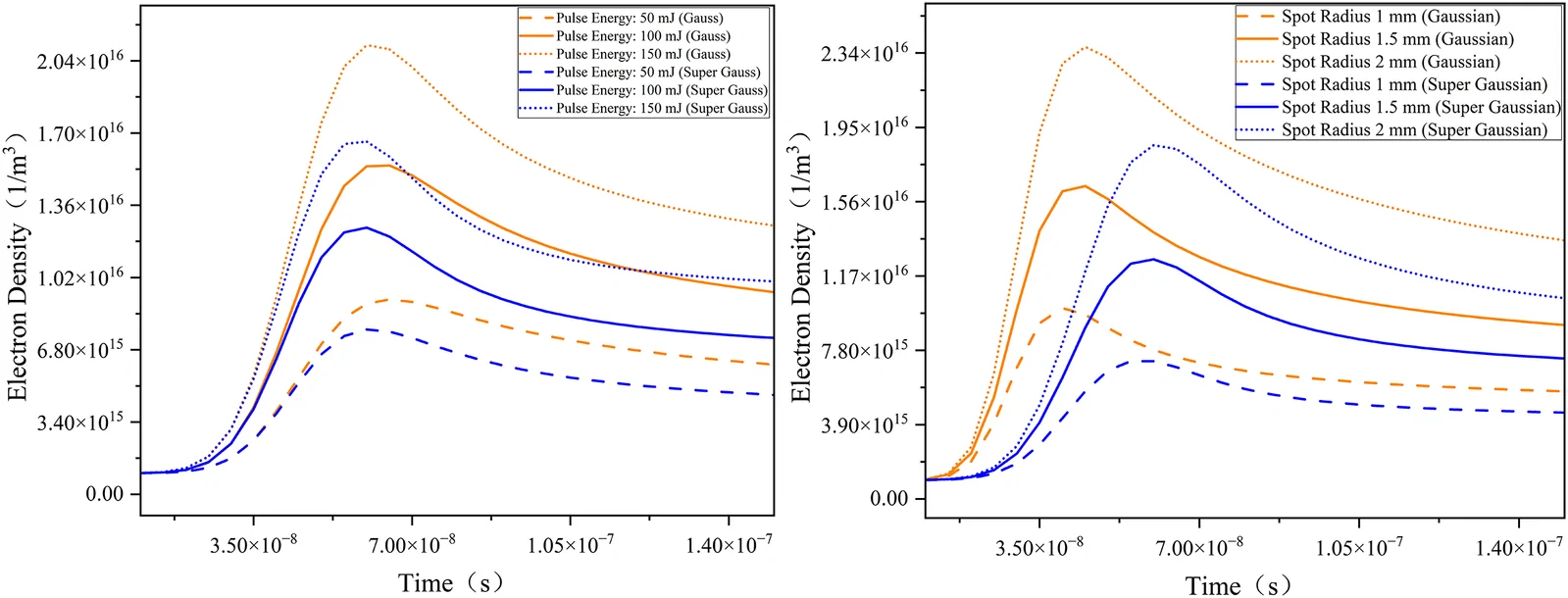
Electron density variation curve.

The electron density of plasma generated by pulsed laser with two pulse shapes under different single pulse energy and spot radius is sorted out, and the curve of electron density changing with time can be obtained, as shown in Fig 9.

In both distributions, the peak electron density increases significantly with the increase of single pulse energy. At the same energy, the electron density generated by the Gaussian distribution is always higher than that of the super-Gaussian distribution. The peak value of electron density increases with the increase of spot radius. The intensity of the Gaussian pulse center is concentrated, and the early ionization region is limited, so the electron density of the Gaussian distribution under the same spot is higher. The energy distribution of the super-Gaussian pulse is uniform, and the initial core density is slightly lower. However, as the spot becomes larger, the energy deposition effect in the peripheral area is better, and the overall density increases slightly faster.

In both pulse-shape distributions, the peak electron density increases markedly with increasing single-pulse energy. At identical energy levels, the Gaussian pulse consistently produces higher electron densities than the super-Gaussian pulse. Peak electron density also increases with spot radius. Due to the strong central intensity of the Gaussian pulse, its early ionization region is more confined, resulting in higher peak electron densities for a given spot radius. In contrast, the super-Gaussian pulse’s uniform energy distribution produces a lower initial core density. However, as the spot size increases, enhanced peripheral energy deposition leads to a comparatively faster rise in overall electron density.

According to the concept of plasma critical density $n_{c}=\omega^{\text{2}}\varepsilon_{\text{0}}m_{e}/e^{\text{2}}$, the laser transmittance drops sharply when the electron density approaches $n_{c}$. Because the peak electron density generated by the super-Gaussian pulse is lower and remains farther from $n_{c}$, the effective transmission window is wider. The spatial broadening of the energy distribution suppresses local ionization and reduces the fraction of laser energy that is shielded by the plasma.

At fixed single-pulse energy and pulse width, increasing the spot radius reduces the peak power density. However, the enlarged spot area increases the irradiated material volume and significantly expands the laser–material interaction region. Thus, although the local field strength decreases, the total ionizable volume increases, raising the initial electron population and enhancing subsequent avalanche-ionization probability. As a result, larger spots yield a greater integrated electron-generation rate, leading to higher peak electron densities.

## Verification experiment of pulsed laser ablation efficiency

To systematically evaluate the influence of different pulse shapes on the laser ablation process, the experimentally measured ablation morphology of 6061 aluminum alloy was compared with multi-physics numerical simulation results under varying single-pulse energies and spot radii [15]. The experimental measurements covered two typical pulse shapes: Gaussian and super-Gaussian. In the experiments, the aluminum alloy surface was ablated at a fixed position under different pulse shapes, single-pulse energies, and spot radii.

By comparing the perforation times obtained from simulations and experiments under different parameter combinations, the accuracy of the numerical model was verified. Five repeated experiments were conducted for each parameter combination, and the final result was obtained by averaging the remaining data after removing the maximum and minimum values. The experimental and simulated results show good consistency in ablation morphology and depth, confirming the validity of the model.

The subtle differences between the simulation and experimental results are primarily attributed to the following factors: (1) the 6061 aluminum alloy samples used in the experiments exhibit microstructural inhomogeneity, whereas the simulation model assumes an ideally homogeneous material; and (2) slight deviations exist between the actual laser pulse and the ideal waveform, particularly in the transient characteristics of the leading and trailing edges of the pulse.

### Experimental preparation and overall layout

**Fig 10:**
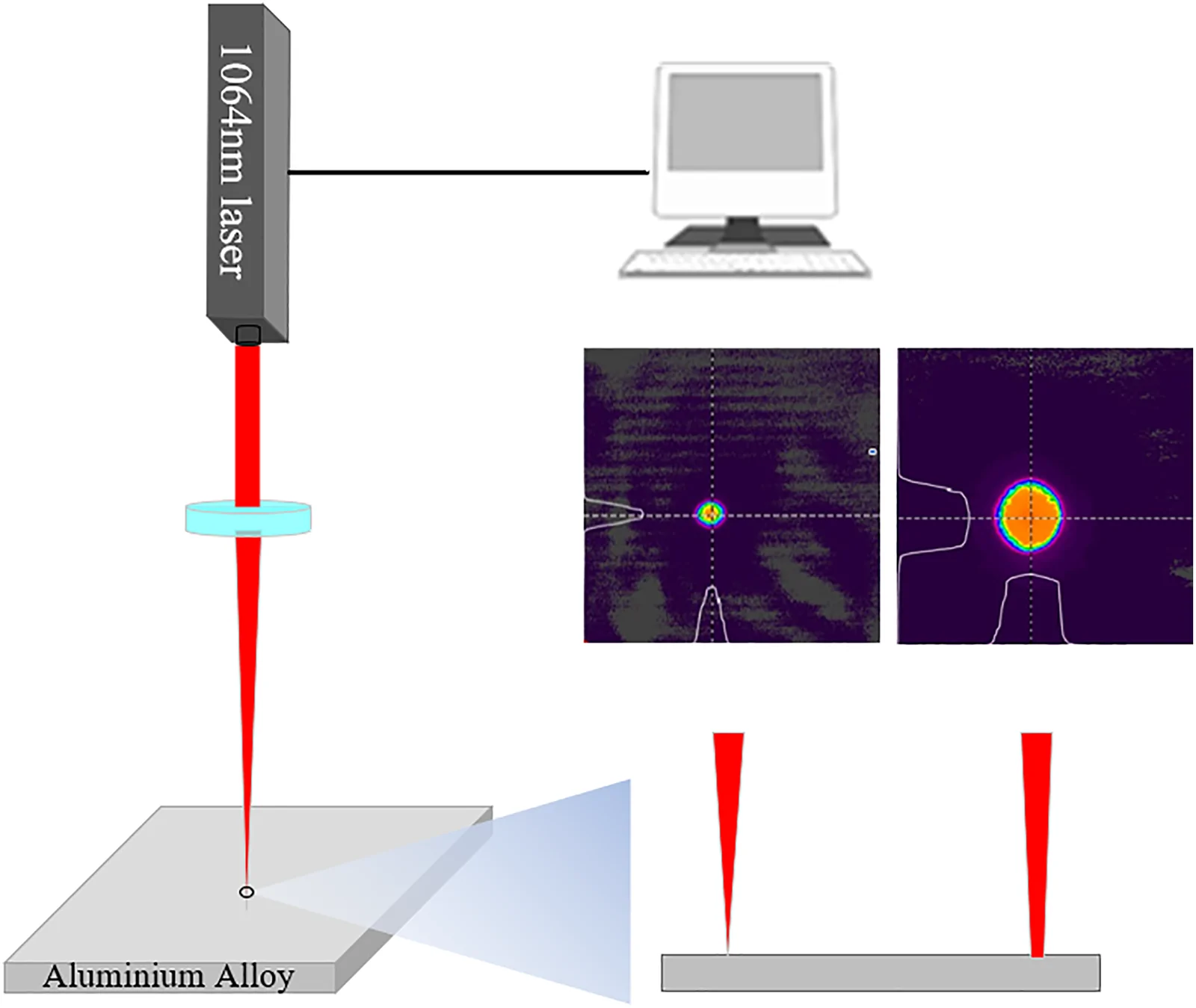
Experimental layout diagram.

**Fig 11:**
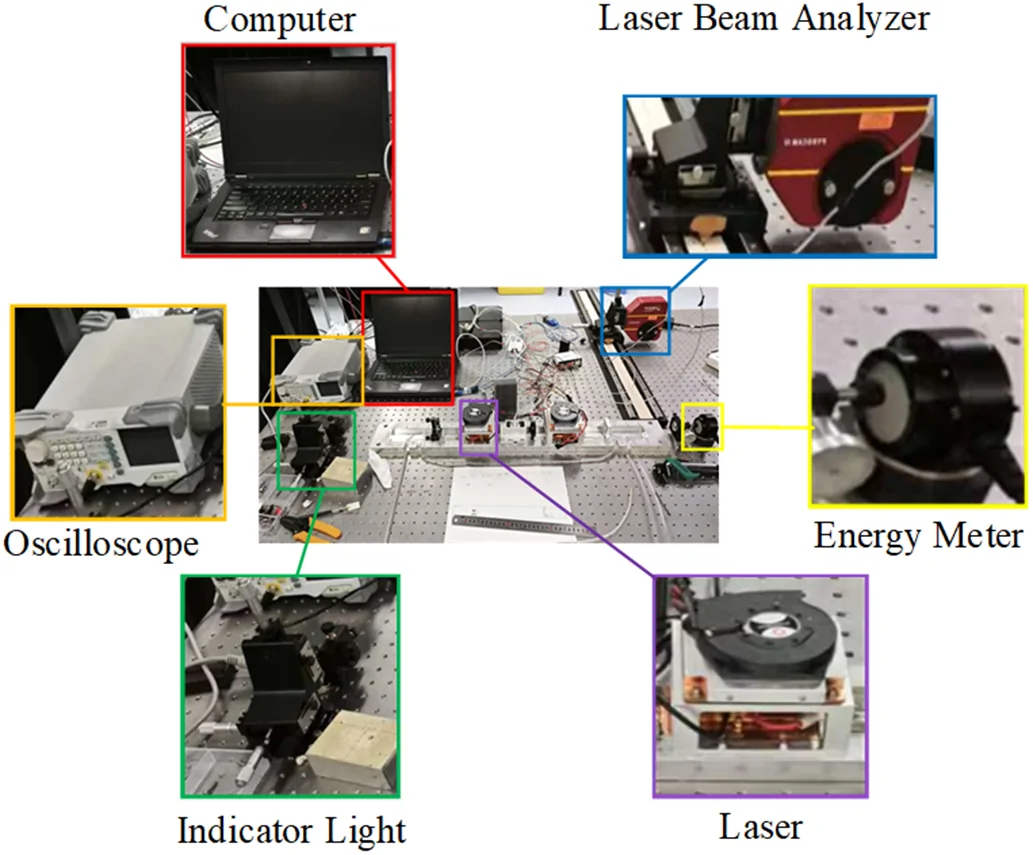
Experimental environment.

The influence of pulse shape on the ablation effect was investigated using a semiconductor laser processing system with a wavelength of 1064nm, as shown in Fig 10. The system integrates a high-power semiconductor laser source whose output is modulated into pulses of different shapes via a pulse generator. After beam shaping, the maximum single-pulse energy available for the ablation experiments is 150 mJ, and the maximum pulse-repetition frequency is 120 Hz. The experimental setup uses a high-power pulsed laser whose waveform and timing are precisely controlled by an acousto-optic modulator. The pulsed laser beam induces ablation on the aluminum alloy plate after being focused by a lens with a focal length of 150 mm. The experimental setup and environment are shown in Fig 11.

**Fig 12:**
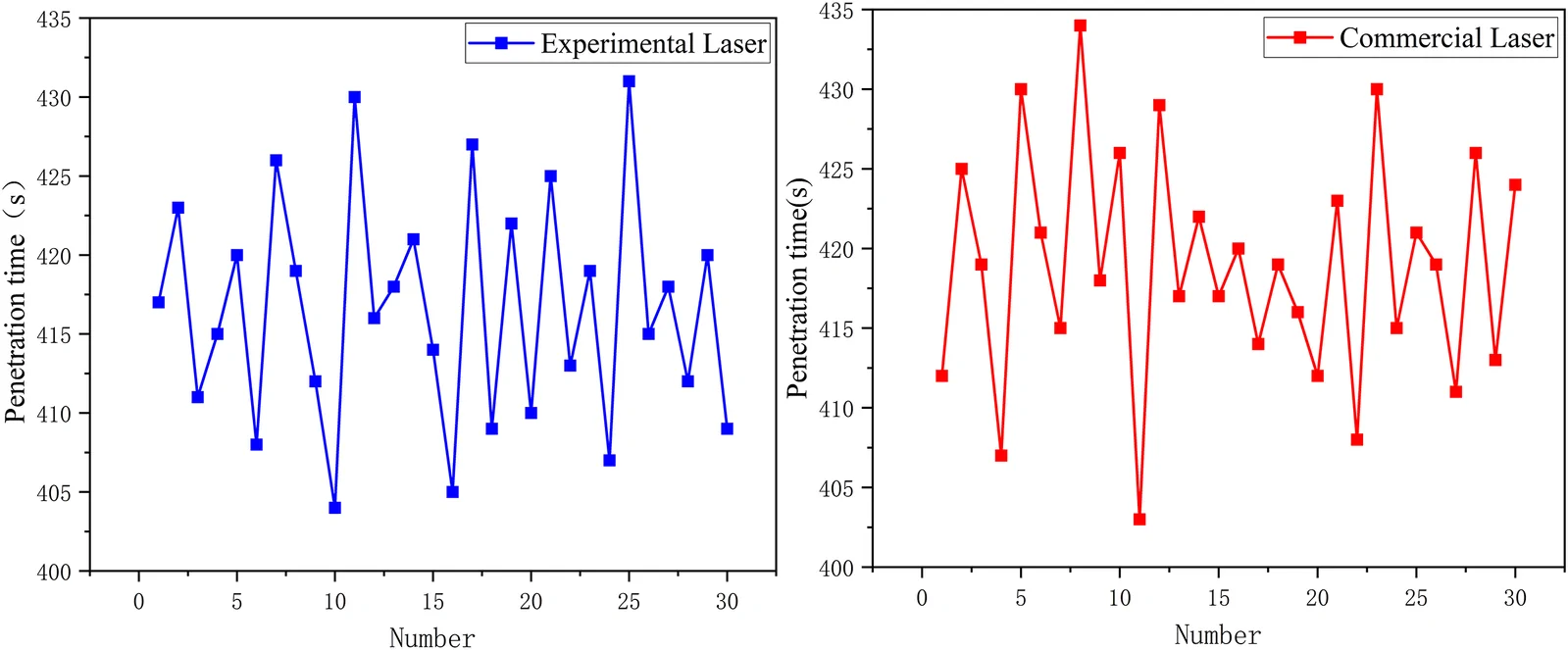
Comparison of ablation time under the same parameters.

To ensure the reliability of the experimental results and to avoid systematic deviations arising from differences in laser performance, the experimental laser was compared with a commercially mature laser. During the experiments, the two lasers were used to ablate aluminum-alloy samples under identical parameters. The single-pulse energies were set to 50 mJ, 100 mJ, and 150 mJ, and 30 repeated ablation trials were conducted at each energy level to obtain statistically meaningful data. The aluminum-alloy ablation results obtained with the two lasers under identical experimental conditions are presented in Fig 12.

**Fig 13:**
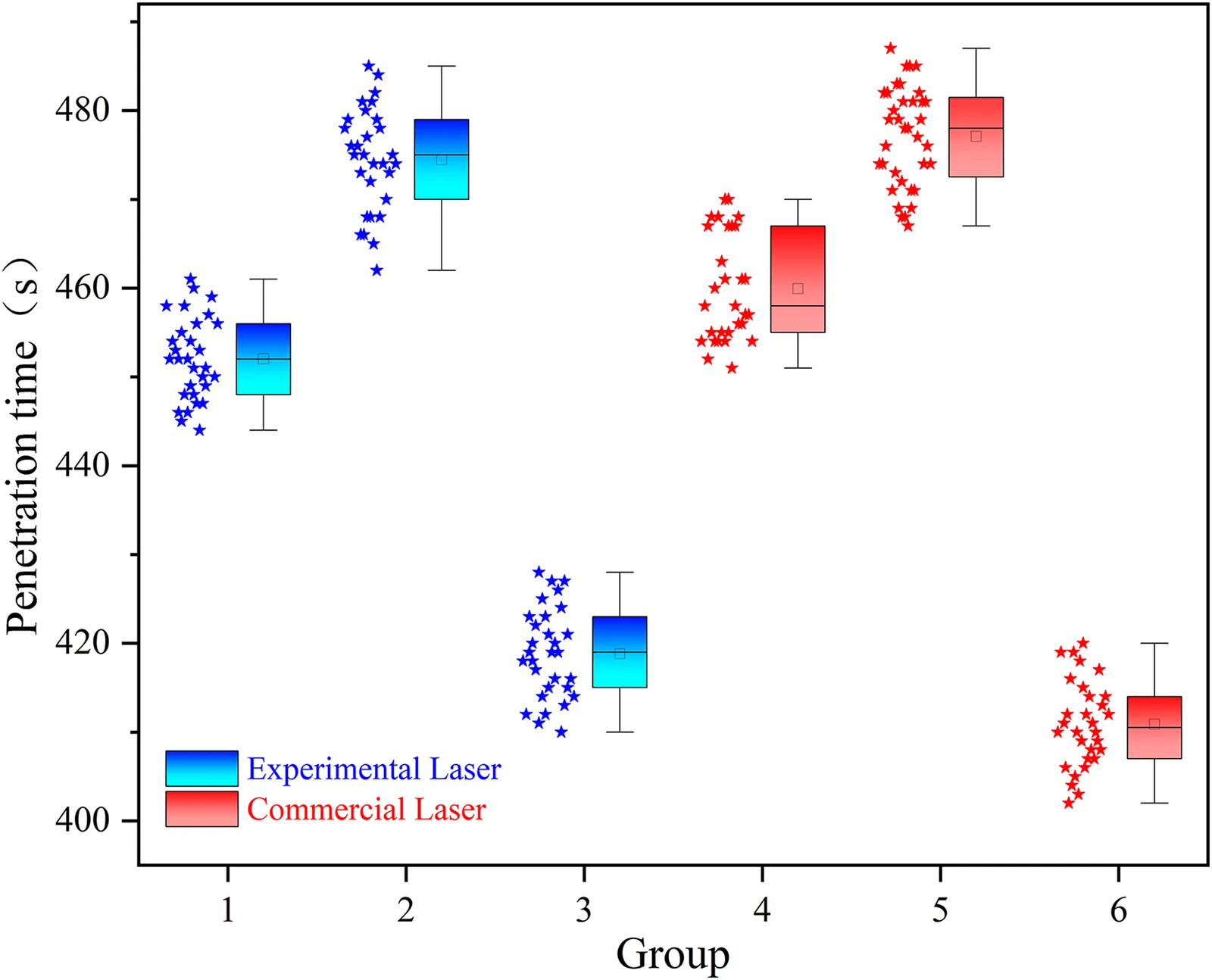
Perforation time comparison box chart.

By recording and analyzing the temporal characteristics of aluminum alloy ablation and perforation induced by two lasers under three single-pulse energy conditions, the experiments were organized into the following groups: (1) experimental laser at 50 mJ,100 mJ, and 150 mJ; and (2) commercial laser at 50 mJ, 100 mJ, and 150 mJ. Statistical analysis was performed using the box plots and dispersion coefficients shown in Fig 13.

Based on the perforation-time records, the dispersion coefficients for each laser under various conditions are presented in Table 5.

**Table 5:** Laser perforation time dispersion coefficient.

	50mJ	100mJ	150mJ
Experimental Laser	1.02%	1.22%	1.20%
Commercial Laser	1.21%	1.20%	1.18%

The results indicate that the ablation performance of the experimental laser is comparable to that of a commercially available, mature laser system. Therefore, the experimental laser demonstrates good stability and repeatability, making it suitable for subsequent investigations into the laser-ablation characteristics of aluminum alloys.

### Experimental scheme and sample description

Based on the experimental optical layout and instrumentation, the protocol was established as follows. As summarized in Table 6, Samples 1–90 were sequentially tested using a 1.5 mm spot radius at single-pulse energies of 50 mJ, 100 mJ, and 150 mJ. Samples 91–180 were subsequently tested at a fixed single-pulse energy of 100 mJ with spot radii of 1 mm, 1.5 mm, and 2 mm. For each condition, the perforation time of the aluminum alloy was recorded, and the perforation performance of Gaussian and super-Gaussian pulses was compared under identical parameters. Each group underwent 30 repeated measurements to ensure statistical robustness.

**Table 6:** Experimental parameters.

Material Type	Sample Serial Number	Pulse Energy(mJ)	Spot Radius(mm)
AL-6061	1-30	50	1.5
	31-60	100	
	61-90	150	
AL-6061	91-120	100	1
	121-150		1.5
	151-180		2

**Fig 14:**
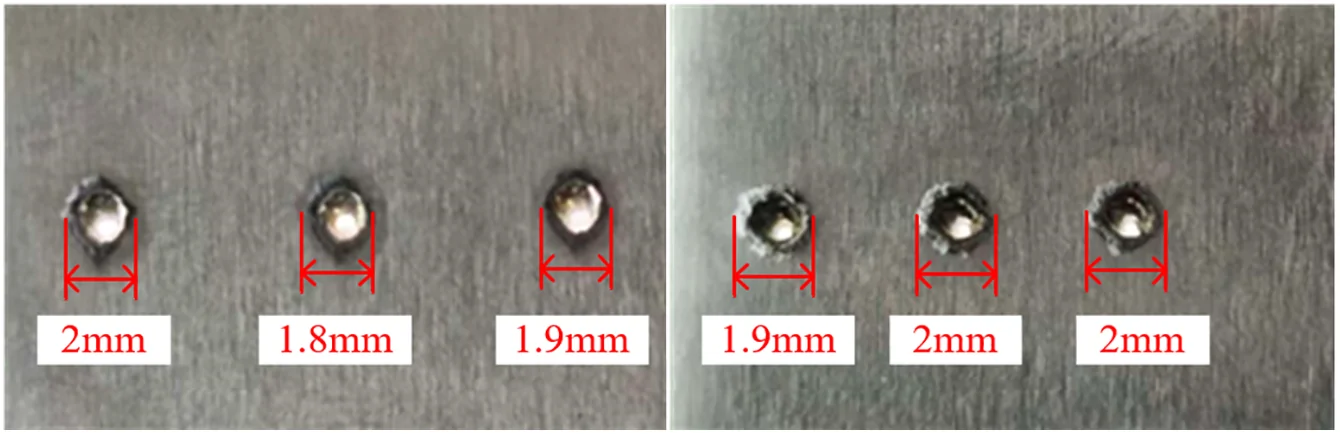
Perforation morphology.

The 6061 aluminum alloy samples were prepared through sequential grinding and polishing prior to the laser ablation experiments. To ensure optimal surface cleanliness, the samples underwent a thorough cleaning procedure consisting of a 10-minute ultrasonic bath in acetone to remove organic contaminants, followed by soaking in anhydrous ethanol for 5 minutes for dehydration, and finally purging and drying with high-purity nitrogen [16]. To systematically investigate the influence of different pulse shapes on the ablation behavior, a series of controlled laser ablation experiments were performed under precisely regulated conditions. The resulting perforation morphology is shown in Fig 14.

### Relationship between pulse shape and perforation time

#### Comparison of perforation time under pulse energy change.

At high energy density, plasma shielding is a non-negligible factor. As the laser energy density increases, the proportion of energy absorbed by the plasma increases significantly. According to the simulation part, the electron density-time curves of Gaussian and super-Gaussian beams at single pulse energy of 50 mJ, 100 mJ and 150 mJ are shown. The plasma absorbs laser energy through inverse bremsstrahlung and photoionization processes. The absorption coefficient $\alpha_{\text{IB}}$ is proportional to the electron density $n_{i}^{\text{2}}$:


$$\begin{array}{c} \alpha_{ib}=(\text{3}.\text{69}\times \text{1}{\text{0}}^{\text{10}}Z^{\text{2}}n_{i}^{\text{2}}/T^{\text{0}.\text{5}}\nu^{\text{3}})[\text{1}-exp(-h\nu /k_{b}T)] \end{array}$$ (8)

As a result, the effective energy delivered to the target surface decreases rather than increases. The additional energy input is primarily consumed to sustain the plasma rather than to remove material, thereby prolonging the perforation time [17]. As the energy density continues to rise, the higher energy deposition causes the target-surface temperature to reach the critical threshold more rapidly, triggering a phase explosion. This nonlinear ablation mechanism produces an extremely high material-removal rate, and its enhancement fully compensates for the plasma-shielding loss, leading to a pronounced reduction in perforation time [18].

**Fig 15:**
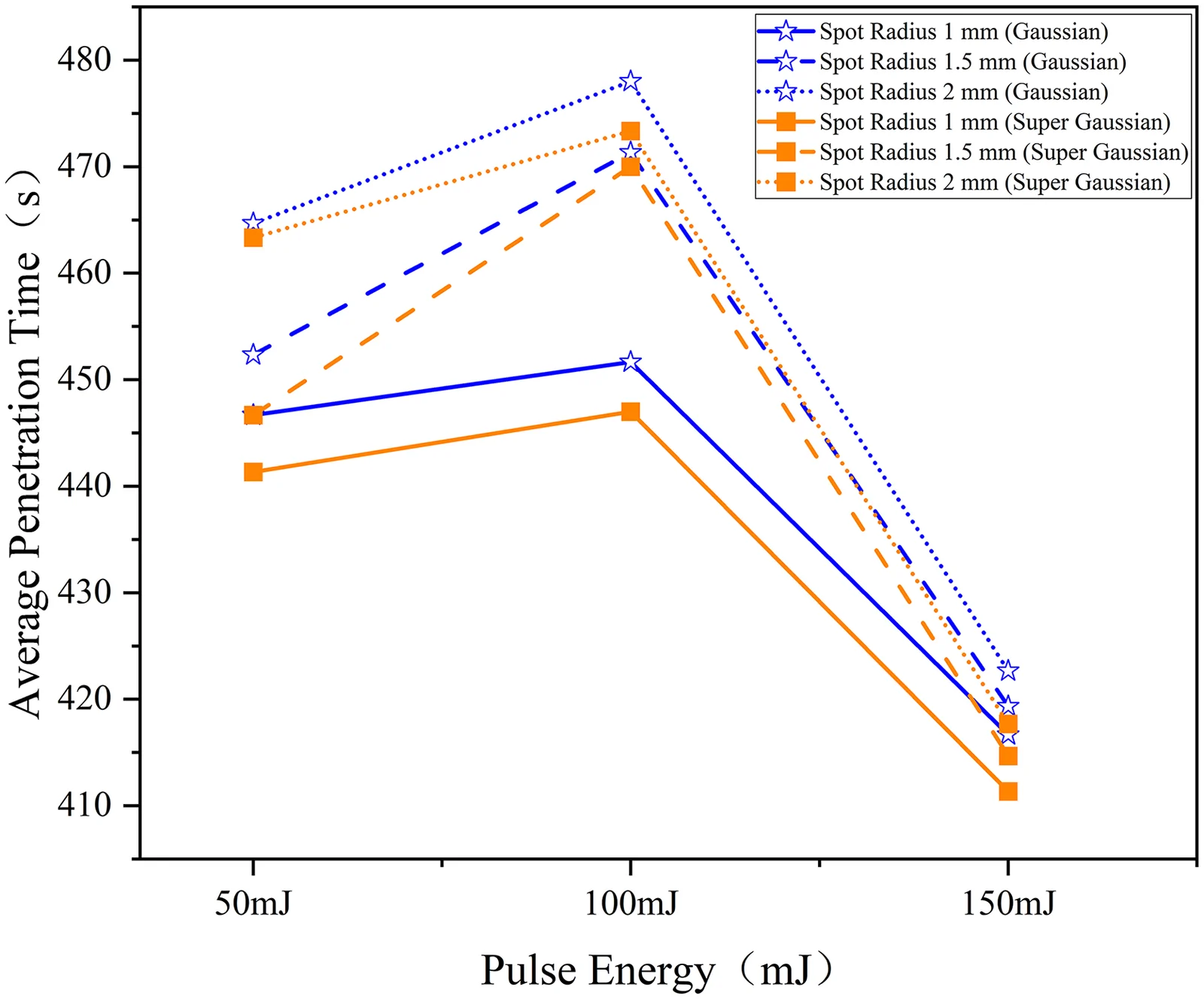
Comparison of perforation time as a function of single-pulse energy.

Under different single-pulse energy conditions, the perforation time of aluminum alloy irradiated by Gaussian and super-Gaussian pulses exhibits an initial increase followed by a decrease, as shown in Fig 15. When the single-pulse energy increases from 50 mJ to 100mJ, the perforation time increases substantially. As the single-pulse energy further increases to 150mJ, the perforation time decreases rapidly, indicating a substantial increase in the perforation rate. In terms of pulse shape, the Gaussian beam is more likely to induce strong plasma shielding because of its high energy concentration, thereby amplifying nonlinear effects. The super-Gaussian beam shows better stability and controllability throughout the machining process owing to its smoother energy distribution [19].

Overall, as the single-pulse energy increases, heat accumulation within the material is enhanced, leading to markedly improved melting and vaporization rates. Pulse-shape modulation effectively alters the energy-deposition profile, which strongly influences the perforation rate and process stability. Super-Gaussian pulses exhibit superior thermal-effect characteristics for achieving efficient perforation owing to their higher energy-utilization efficiency and more uniform power distribution.

#### Comparison of perforation time under the change of spot radius.

During the laser ablation process, the laser pulse typically persists for a relatively long duration. When the laser interacts with the material, it vaporizes the surface and generates a cluster of high-temperature, high-pressure plasma and gaseous species. This plume expands upward from the material surface during the laser pulse. The energy of subsequent laser pulses must propagate through this plume before reaching the material surface. Particles within the plume absorb and scatter the incoming laser energy, leading to a reduction in the energy that effectively reaches the material surface.

After the laser passes through the plume, the energy density reaching the material surface can be described by Lambert–Beer’s law:


$$\begin{array}{c} F_{\text{eff}}=F_{\text{0}}exp(-\alpha \rho_{p}h) \end{array}$$ (9)

According to the conservation of mass, the ablation mass is equal to the plume mass, and the relationship between plume density and radius can be obtained [20]:


$$\begin{array}{c} \rho_{p}=\frac{\pi \frac{d^{\text{2}}}{\text{4}}T\rho_{b}}{V_{p}} \end{array}$$ (10)

The plume density is brought into the ablation efficiency formula to obtain:


$$\begin{array}{c} T=Kln\left(\frac{F_{\text{0}}exp(-\alpha \rho_{p}h)}{F_{t}}\right)=K\left[ln\left(\frac{F_{\text{0}}}{F_{t}}\right)-\alpha \rho_{p}h\right] \end{array}$$ (11)

The hitting dose SD is the number of pulses required to penetrate the sample with a thickness of L. Taking the hitting dose as the relationship between the spot radius and the ablation efficiency [21], it can be obtained:


$$\begin{array}{c} SD=\frac{L}{T_{\text{0}}}+\frac{A\cdot d^{\text{2}}}{d^{\text{2}}+G\cdot d+G^{\text{2}}/\text{3}} \end{array}$$ (12)

For small laser spots, the lateral expansion scale of the plume approaches a quasi-constant background. The ablated material is therefore diluted within a nearly fixed plume volume. Consequently, a smaller spot produces less ablated mass, results in a lower plume density and a weaker shielding effect, and ultimately leads to significantly enhanced effective energy delivery and ablation efficiency.

**Fig 16:**
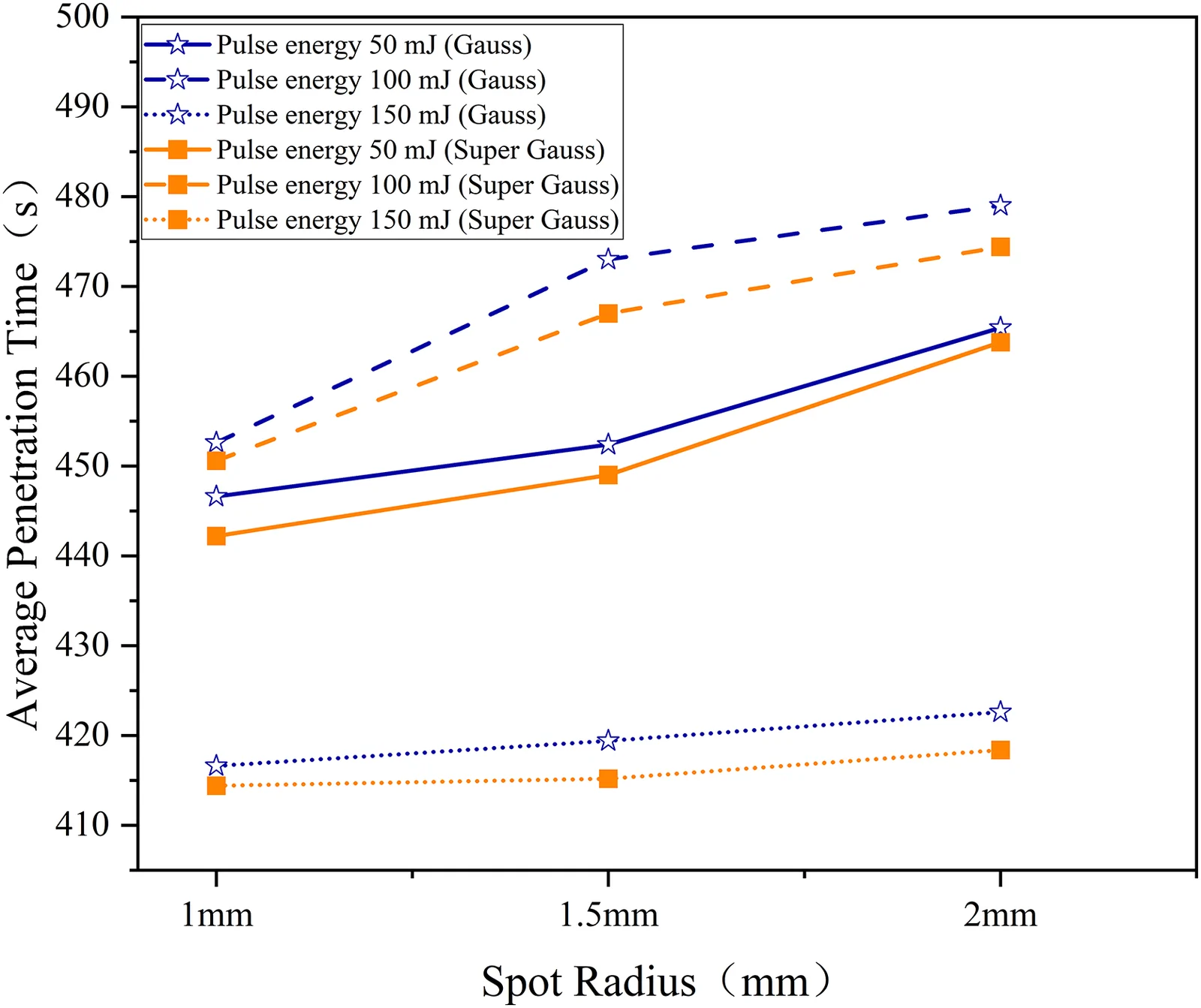
Comparison of perforation time under the change of spot radius.

Fig 16 shows the mean perforation time and its associated error for the Gaussian and super-Gaussian beams irradiating the aluminum alloy target at different spot radii under the same single-pulse energy condition. The perforation time of the Gaussian beam initially increases and then decreases slightly as the spot radius increases, whereas that of the super-Gaussian beam increases more gradually with increasing spot radius. The perforation time of the super-Gaussian beam is consistently shorter than that of the Gaussian beam at the same spot radius.

Under the same single-pulse energy, the perforation times of the Gaussian and super-Gaussian beams are influenced by variations in spot radius. For the Gaussian beam, increasing the spot radius from 1 mm to 2 mm significantly prolongs the perforation time, indicating that the reduction in energy density decreases the material-heating and gasification rates. When the radius is further increased to 3 mm, the perforation time decreases slightly, likely due to edge-energy effects and localized reheating of the material.

In contrast, the perforation time of the super-Gaussian beam varies more smoothly with radius, and its value at 1 mm is significantly lower than that of the Gaussian beam, suggesting that the flat-topped energy profile enhances energy utilization and thermal uniformity. The error bars show that the Gaussian beam exhibits large fluctuations at small radii, whereas the super-Gaussian beam demonstrates better overall stability. Overall, the super-Gaussian beam exhibits higher energy-coupling efficiency and greater perforation stability under the same single-pulse energy, particularly at small spot sizes.

## Discussion

The superior performance of super-Gaussian pulses stems from their flat-top energy distribution, which addresses key limitations of Gaussian pulses:

(1) Uniform Energy Deposition: Reduces localized overheating and HAZ, improving processing precision.
(2) Mitigated Plasma Shielding: Lower peak electron density extends laser transmission, enhancing energy-coupling efficiency by 3–18%.
(3) Stable Perforation: Consistent energy delivery minimizes process variability, critical for high-precision manufacturing.

Single-pulse energy and spot radius also play synergistic roles: higher energy increases ablation depth but intensifies plasma shielding, while larger spot radii reduce energy density but expand the laser-material interaction volume. Super-Gaussian pulses balance these effects, maintaining efficiency across a broader parameter range.

This work provides a validated framework for pulse shape optimization, complementing existing studies on pulse width and energy density. The findings are particularly relevant for lightweight metal processing in aerospace and automotive industries, where precision and efficiency are paramount.

The superior performance of super‑Gaussian pulses originates from their flat‑top energy distribution, which addresses three key limitations of Gaussian pulses:

(1) Uniform energy deposition and plasma shielding mitigation.

The peak electron density for Gaussian pulses is 1.7–2.4 times higher than that of super‑Gaussian pulses (Tables 3 and 4). According to Eq. (4), the inverse‑bremsstrahlung absorption coefficient $\alpha_{IB}$ scales linearly with $n_{e}$. Using the simulated electron‑density profiles, we estimate that, at 150 mJ and 2 mm spot radius, roughly 30% of the incident laser energy is absorbed by the plasma for Gaussian pulses, whereas only ~18% is absorbed for super‑Gaussian pulses. This directly translates into the observed 3–18% improvement in effective energy‑coupling efficiency, with the largest gains occurring near the ablation threshold where plasma shielding is moderate.

(2) Melt‑pool dynamics and microstructural implications.

Although our model does not include fluid flow, the computed temperature gradients provide insight into melt‑pool dimensions and lifetime. Super‑Gaussian pulses create a broader, shallower melt pool with a longer residence time in the liquid state, which may promote grain coarsening in the recast layer. Conversely, Gaussian pulses produce a deeper, narrower melt pool with rapid quenching, potentially leading to finer recast grains but also to higher residual stresses. These predictions are consistent with metallographic studies on laser‑drilled aluminum (e.g., Tan et al., 2018) and suggest that pulse shape can be tailored to control the recast layer characteristics.

(3) Practical relevance and limitations.

For industrial laser drilling, the reduction of plasma shielding by super‑Gaussian pulses not only shortens perforation time but also improves hole‑quality consistency, as evidenced by the lower dispersion coefficients. However, the benefit diminishes at extremely high fluences where plasma shielding becomes dominant for both pulse shapes. Future work should incorporate multiphysics simulation of melt flow and vapor dynamics to quantitatively predict recast layer thickness and composition. Additionally, in‑process monitoring of plasma emission could provide real‑time feedback for adaptive pulse shaping.

## Conclusion

In this study, the effects of pulse shape, single-pulse energy, and spot radius on the laser ablation behavior of 6061 aluminum alloy were systematically investigated using finite-element numerical simulations and controlled-variable experiments. Within the energy range of 50–150 mJ and spot radius 1–2 mm, the super‑Gaussian pulse consistently produces a greater ablation depth than the Gaussian pulse. Its flat‑top energy distribution effectively weakens plasma shielding, resulting in a 3–18% increase in energy‑utilization efficiency. This range is in good agreement with the 5–20% efficiency gains reported for beam‑shaping techniques in metal ablation

The experimentally measured perforation time shows good agreement with the numerical predictions. The maximum difference in the perforation-time dispersion coefficient between the experimental laser and a commercially available laser is only 0.19%, confirming the reliability of the experimental system. The perforation time first increases and then decreases with increasing pulse energy, corresponding to the electron-density turning point predicted by the simulations. Overall, the super-Gaussian pulse exhibits a lower and more stable perforation time across all energy and spot-size conditions, and the maximum difference in average perforation time between the two pulse shapes is approximately 6 s. This study provides a physically consistent and experimentally supported evaluation of the effect of pulse shaping on the ablation efficiency of high-energy laser. The research results highlight the practical significance of pulse space shaping as an effective means to regulate energy deposition and improve processing stability in the application of laser-metal interaction.
