# Supplementary material for: High-power pulsed-laser ablation of aluminum alloy: Simulation model and experimental validation
Source: PLoS One. 2026 Sep 3;21(9):e0357262. doi: 10.1371/journal.pone.0357262 (PMC13541167; doi:10.1371/journal.pone.0357262)
Supplement: Supplementary file 1 [file pone.0357262.s001.docx]

Dear Editor and Reviewers,

We sincerely thank the reviewers for their careful reading and insightful comments. These suggestions have been invaluable in improving the quality of our manuscript. Below, we provide a detailed point-by-point response to each comment, indicating the exact changes made in the revised manuscript.

Reviewer #1

Comment 1: The English writing needs to be improved.

Response: Thank you for pointing this out. The entire manuscript has been thoroughly proofread and edited. Numerous grammatical errors, awkward phrasing, and inconsistencies in writing style have been corrected. All revisions have now been incorporated into the text, thereby enhancing the article’s overall readability and academic quality.

Comment 2: The chemical elements and mechanical properties for the material 6061 aluminum alloy should be added.

Response: A new table (Table 2) has been added in Section 3.2, listing the typical chemical composition (Si, Fe, Cu, Mn, Mg, Cr, Zn, Ti, Al) and key mechanical properties (tensile strength, yield strength, elongation, hardness) of 6061 aluminum alloy.

Comment 3: The information for the finite element model is missing, like type of element? Number of elements and nodes?

Response: We have substantially expanded the description of the finite-element model in Sections 3.1 and 3.2.

Comment 4: What is the unit in Eq(5) for parameter T?

Response: We apologize for the omission. In the revised manuscript, the temperature 𝑇 in the thermal-diffusion equation (now Eq. 2) is explicitly stated to be in Kelvin (K). All units throughout the manuscript have been double-checked to avoid ambiguity.

Comment 5:  In Fig. 4, there is Chinese character.

Response: Figure 4 has been updated – all Chinese labels have been replaced with their English equivalents.

Comment 6: “Gaussian and super-Gaussian beams were used as the laser heat sources.” Are there any other types of beams suitable?

Response: Two representative pulse shapes, Gaussian and super-Gaussian, were selected for this study because they exemplify the typical bell-shaped and flat‑top energy distributions, respectively. Other pulse shapes (e.g., triangular or arbitrary waveforms) are beyond the scope of the present work.

Comment 7: “The pulsed laser was assumed to be normally incident at the center of the aluminum-alloy surface.” Is there any reference?

Response: A justification has been added, citing standard modelling practice. The text now reads: “Following common practice in laser-ablation modeling [15], the pulsed laser is assumed to be normally incident at the center of the aluminum-alloy surface.” Reference [15] has been verified to support this assumption.

Comment 8: The discussion is not enough, you should explain more useful information.

Response: The Discussion (Section 5) has been completely rewritten and extended. It now provides: A quantitative analysis of plasma shielding and energy‑coupling efficiency, estimating that up to 30% of laser energy can be shielded by the plasma for Gaussian pulses, compared to 15–20% for super-Gaussian pulses.An interpretation of how the temperature distribution induced by different pulse shapes influences melt‑pool lifetime and recast‑layer formation.Practical guidelines for industrial laser processing: super-Gaussian pulses are recommended for high‑aspect‑ratio drilling due to lower plasma losses, whereas Gaussian pulses may be advantageous for surface texturing where sharp thermal gradients are desired.A discussion of the limitations of the present model and an outlook for future work including fluid dynamics and experimental microstructure characterization.

Reviewer #2

Comment 1: The second chapter, the theoretical model section, provides insufficient details on the construction of the finite element model. For example: The boundary conditions (such as thermal boundary, convection, radiation, etc.) have not been clearly defined; the method of grid division and convergence analysis have not been explained; the type of solver, time step, and nonlinear processing method have not been provided.

Response: We fully agree with this constructive remark. All of the requested details have now been incorporated into Sections 2 and 3:Boundary conditions are explicitly listed (see response to Reviewer #1, Comment 3).Meshing and convergence analysis are described in Section 3.1, including the element type, element count, refinement strategy, and convergence test results. Solver settings are now provided: time‑dependent study with adaptive time stepping, on the residual; phase‑change handled by the enthalpy method; material removal implemented by deactivating elements whose temperature exceeds the boiling point

Comment 2: The content of Part 5, "Discussion", is overly repetitive with the results section and lacks in-depth analysis of the underlying mechanisms.Further exploration is needed on the quantitative relationship between plasma shielding and energy coupling, the influence of different pulse shapes on the molten pool flow and recrystallization structure, as well as the gap and promotion path between these findings and actual industrial applications.

Response: We appreciate this insightful suggestion. The Discussion section has been thoroughly restructured to focus on mechanistic insight rather than summarizing results. Plasma shielding—quantitative relationship of energy coupling: Based on simulation-derived curves of electron density versus time, we estimated the proportion of laser energy absorbed by the plasma using the effective absorption coefficient. This analysis directly explains the 3–18% improvement in energy utilization efficiency observed for ultra-high-Gauss pulses. Although the current model does not account for the fluid dynamics of melt flow, we inferred potential microstructural effects using computationally derived temperature gradients and melt pool lifetime. Literature data on similar alloys were cited to support these qualitative correlations. A new section has been added to bridge the gap between laboratory research and actual manufacturing processes. It explores how optimizing pulse shape can reduce the scrap rate in laser drilling of aerospace components and emphasizes the necessity of online monitoring of plasma emission.

Comment 3: The conclusion section lacks quantitative comparison.Although it is mentioned that "ultra-Gaussian pulses increase energy utilization by approximately 3–18%", no comparison with the literature or description of the applicable range is provided. Suggestion: Add quantitative comparisons of different pulse shapes from existing studies to enhance the persuasiveness of the conclusion.

Response: The Conclusions section has been reinforced by incorporating quantitative comparisons from the recent literature. For instance, studies on beam shaping in metal ablation (e.g., Bakhtari et al., 2024) report efficiency enhancements of 5–20%, which aligns well with our 3–18% range. The applicable parameter window (energy 50–150 mJ, spot radius 1–2 mm) is now explicitly stated, and it is noted that the advantage of super‑Gaussian pulses is most pronounced near the ablation threshold where plasma shielding is relatively weak.

We believe that these revisions fully address all concerns raised by the reviewers. The manuscript has been significantly improved in both depth and clarity. We hope it is now acceptable for publication in PLOS ONE.

Sincerely,

The authors
