# Supplementary material for: High-power pulsed-laser ablation of aluminum alloy: Simulation model and experimental validation
Source: PLoS One. 2026 Sep 3;21(9):e0357262. doi: 10.1371/journal.pone.0357262 (PMC13541167; doi:10.1371/journal.pone.0357262)
Supplement: Peer review history

# Peer review: Decision Letter 0

**DOI:** 10.1371/journal.pone.0357262.r001

**Authors:** Khalil Abdelrazek Khalil

9 Jun 2026

Dear Dr. hou,

Thank you for submitting your manuscript to PLOS ONE. After careful consideration, we feel that it has merit but does not fully meet PLOS ONE’s publication criteria as it currently stands. Therefore, we invite you to submit a revised version of the manuscript that addresses the points raised during the review process.

Please submit your revised manuscript by Jul 24 2026 11:59PM. If you will need more time than this to complete your revisions, please reply to this message or contact the journal office at plosone@plos.org. When you're ready to submit your revision, log on to https://www.editorialmanager.com/pone/ and select the 'Submissions Needing Revision' folder to locate your manuscript file.

- A letter that responds to each point raised by the academic editor and reviewer(s). You should upload this letter as a separate file labeled 'Response to Reviewers'.
- A marked-up copy of your manuscript that highlights changes made to the original version. You should upload this as a separate file labeled 'Revised Manuscript with Track Changes'.
- An unmarked version of your revised paper without tracked changes. You should upload this as a separate file labeled 'Manuscript'.

If you would like to make changes to your financial disclosure, please include your updated statement in your cover letter. Guidelines for resubmitting your figure files are available below the reviewer comments at the end of this letter.

If applicable, we recommend that you deposit your laboratory protocols in protocols.io to enhance the reproducibility of your results. Protocols.io assigns your protocol its own identifier (DOI) so that it can be cited independently in the future. For instructions see: https://journals.plos.org/plosone/s/submission-guidelines#loc-laboratory-protocols. Additionally, PLOS ONE offers an option for publishing peer-reviewed Lab Protocol articles, which describe protocols hosted on protocols.io. Read more information on sharing protocols at https://plos.org/protocols?utm_medium=editorial-email&utm_source=authorletters&utm_campaign=protocols.

As the corresponding author, your ORCID iD is verified in the submission system and will appear in the published article. PLOS supports the use of ORCID, and we encourage all coauthors to register for an ORCID iD and use it as well. Please encourage your coauthors to verify their ORCID iD within the submission system before final acceptance, as unverified ORCID iDs will not appear in the published article. *Only*  the individual author can complete the verification step; PLOS staff *cannot*  verify ORCID iDs on behalf of authors.

We look forward to receiving your revised manuscript.

Kind regards,

Khalil Abdelrazek Khalil, Ph.D.

Academic Editor

PLOS One

**Journal Requirements:**

When submitting your revision, we need you to address these additional requirements.

1. Please ensure that your manuscript meets PLOS ONE's style requirements, including those for file naming. The PLOS ONE style templates can be found at

https://journals.plos.org/plosone/s/file?id=wjVg/PLOSOne_formatting_sample_main_body.pdf and

https://journals.plos.org/plosone/s/file?id=ba62/PLOSOne_formatting_sample_title_authors_affiliations.pdf

2. Please note that PLOS One has specific guidelines on code sharing for submissions in which author-generated code underpins the findings in the manuscript. In these cases, we expect all author-generated code to be made available without restrictions upon publication of the work. Please review our guidelines at https://journals.plos.org/plosone/s/materials-and-software-sharing#loc-sharing-code and ensure that your code is shared in a way that follows best practice and facilitates reproducibility and reuse.

3. We note that the grant information you provided in the ‘Funding Information’ and ‘Financial Disclosure’ sections do not match.

When you resubmit, please ensure that you provide the correct grant numbers for the awards you received for your study in the ‘Funding Information’ section.

4. Thank you for stating the following financial disclosure:

“National Science and Technology Major Project”

Please state what role the funders took in the study.  If the funders had no role, please state: "The funders had no role in study design, data collection and analysis, decision to publish, or preparation of the manuscript."

If this statement is not correct you must amend it as needed.

Please include this amended Role of Funder statement in your cover letter; we will change the online submission form on your behalf.

5. Please note that funding information should not appear in any section or other areas of your manuscript. We will only publish funding information present in the Funding Statement section of the online submission form. Please remove any funding-related text from the manuscript.

6. We note that your Data Availability Statement is currently as follows:

“All relevant data are within the manuscript and its Supporting Information files.”

Please confirm at this time whether or not your submission contains all raw data required to replicate the results of your study. Authors must share the “minimal data set” for their submission. PLOS defines the minimal data set to consist of the data required to replicate all study findings reported in the article, as well as related metadata and methods (https://journals.plos.org/plosone/s/data-availability#loc-minimal-data-set-definition).

For example, authors should submit the following data:

- The values behind the means, standard deviations and other measures reported;

- The values used to build graphs;

- The points extracted from images for analysis.

Authors do not need to submit their entire data set if only a portion of the data was used in the reported study.

If your submission does not contain these data, please either upload them as Supporting Information files or deposit them to a stable, public repository and provide us with the relevant URLs, DOIs, or accession numbers. For a list of recommended repositories, please see https://journals.plos.org/plosone/s/recommended-repositories.

If there are ethical or legal restrictions on sharing a de-identified data set, please explain them in detail (e.g., data contain potentially sensitive information, data are owned by a third-party organization, etc.) and who has imposed them (e.g., an ethics committee). Please also provide contact information for a data access committee, ethics committee, or other institutional body to which data requests may be sent. If data are owned by a third party, please indicate how others may request data access.

7. We note that Figure 11 and 14 in your submission contain copyrighted images. All PLOS content is published under the Creative Commons Attribution License (CC BY 4.0), which means that the manuscript, images, and Supporting Information files will be freely available online, and any third party is permitted to access, download, copy, distribute, and use these materials in any way, even commercially, with proper attribution. For more information, see our copyright guidelines: http://journals.plos.org/plosone/s/licenses-and-copyright.

We require you to either (1) present written permission from the copyright holder to publish these figures specifically under the CC BY 4.0 license, or (2) remove the figures from your submission:

1. You may seek permission from the original copyright holder of Figures 11 and 14 to publish the content specifically under the CC BY 4.0 license.

We recommend that you contact the original copyright holder with the Content Permission Form (http://journals.plos.org/plosone/s/file?id=7c09/content-permission-form.pdf) and the following text:

“I request permission for the open-access journal PLOS ONE to publish XXX under the Creative Commons Attribution License (CCAL) CC BY 4.0 (http://creativecommons.org/licenses/by/4.0/). Please be aware that this license allows unrestricted use and distribution, even commercially, by third parties. Please reply and provide explicit written permission to publish XXX under a CC BY license and complete the attached form.”

Please upload the completed Content Permission Form or other proof of granted permissions as an "Other" file with your submission.

In the figure caption of the copyrighted figure, please include the following text: “Reprinted from [ref] under a CC BY license, with permission from [name of publisher], original copyright [original copyright year].”

2. If you are unable to obtain permission from the original copyright holder to publish these figures under the CC BY 4.0 license or if the copyright holder’s requirements are incompatible with the CC BY 4.0 license, please either i) remove the figure or ii) supply a replacement figure that complies with the CC BY 4.0 license. Please check copyright information on all replacement figures and update the figure caption with source information. If applicable, please specify in the figure caption text when a figure is similar but not identical to the original image and is therefore for illustrative purposes only.

8. We note you have included a table to which you do not refer in the text of your manuscript. Please ensure that you refer to Tables 2 and 7 in your text; if accepted, production will need this reference to link the reader to the Table.

9. Please include a copy of Table 4 which you refer to in your text on page 9.

10. If the reviewer comments include a recommendation to cite specific previously published works, please review and evaluate these publications to determine whether they are relevant and should be cited. There is no requirement to cite these works unless the editor has indicated otherwise.

[Note: HTML markup is below. Please do not edit.]

Reviewers' comments:

Reviewer's Responses to Questions

**Comments to the Author**

1. Is the manuscript technically sound, and do the data support the conclusions?

Reviewer #1: Yes

Reviewer #2: Yes

**********

2. Has the statistical analysis been performed appropriately and rigorously?

Reviewer #1: Yes

Reviewer #2: Yes

**********

3. Have the authors made all data underlying the findings in their manuscript fully available?

The PLOS Data policy

Reviewer #1: Yes

Reviewer #2: Yes

**********

4. Is the manuscript presented in an intelligible fashion and written in standard English?

Reviewer #1: Yes

Reviewer #2: Yes

**********

Reviewer #1: This paper focuses on the high-power pulsed-laser ablation of aluminum alloy based on the numerical simulation and experimental work. It is interesting, however, here are some suggestions:

1. The English writing needs to be improved.

2. The chemical elements and mechanical properties for the material 6061 aluminum alloy should be added.

3. The information for the finite element model is missing, like type of element? Number of elements and nodes?

4. What is the unit in Eq(5) for parameter T?

5. In Fig. 4, there is Chinese character.

6. “Gaussian and super-Gaussian beams were used as the laser heat sources.” Are there any other types of beams suitable?

7. “The pulsed laser was assumed to be normally incident at the center of the aluminum-alloy surface.” Is there any reference?

8. The discussion is not enough, you should explain more useful information.

Reviewer #2: This paper systematically investigates the influence of Gaussian pulses and super-Gaussian pulses on the laser ablation behavior of 6061 aluminum alloy. Combining finite element numerical simulation with experimental verification, this study analyzes the temperature field, plasma electron density and ablation efficiency under different pulse energies and spot radii. The research findings demonstrate that super-Gaussian pulses exhibit higher energy coupling efficiency and material removal efficiency owing to their more uniform energy distribution. Overall, this research possesses substantial engineering application value, with sound methodology, detailed data and clear structure. Nevertheless, there remains room for improvement in model details, theoretical depth, linguistic presentation and the analysis of partial data.

1. The second chapter, the theoretical model section, provides insufficient details on the construction of the finite element model. For example: The boundary conditions (such as thermal boundary, convection, radiation, etc.) have not been clearly defined; the method of grid division and convergence analysis have not been explained; the type of solver, time step, and nonlinear processing method have not been provided.

2. The content of Part 5, "Discussion", is overly repetitive with the results section and lacks in-depth analysis of the underlying mechanisms.Further exploration is needed on the quantitative relationship between plasma shielding and energy coupling, the influence of different pulse shapes on the molten pool flow and recrystallization structure, as well as the gap and promotion path between these findings and actual industrial applications.

3. The conclusion section lacks quantitative comparison.Although it is mentioned that "ultra-Gaussian pulses increase energy utilization by approximately 3–18%", no comparison with the literature or description of the applicable range is provided. Suggestion: Add quantitative comparisons of different pulse shapes from existing studies to enhance the persuasiveness of the conclusion.

**********

what does this mean?). If published, this will include your full peer review and any attached files.

If you choose “no”, your identity will remain anonymous but your review may still be made public.

**Do you want your identity to be public for this peer review?**  For information about this choice, including consent withdrawal, please see our Privacy Policy

Reviewer #1: No

Reviewer #2: No

**********

[NOTE: If reviewer comments were submitted as an attachment file, they will be attached to this email and accessible via the submission site. Please log into your account, locate the manuscript record, and check for the action link "View Attachments". If this link does not appear, there are no attachment files.]

To ensure your figures meet our technical requirements, please review our figure guidelines: https://journals.plos.org/plosone/s/figures

You may also use PLOS’s free figure tool, NAAS, to help you prepare publication quality figures: https://journals.plos.org/plosone/s/figures#loc-tools-for-figure-preparation.

NAAS will assess whether your figures meet our technical requirements by comparing each figure against our figure specifications.

# Author comment: Author response to Decision Letter 1

**DOI:** 10.1371/journal.pone.0357262.r002

12 Aug 2026

Dear Editor and Reviewers,

We sincerely thank the reviewers for their careful reading and insightful comments. These suggestions have been invaluable in improving the quality of our manuscript. Below, we provide a detailed point-by-point response to each comment, indicating the exact changes made in the revised manuscript.

Reviewer #1

Comment 1: The English writing needs to be improved.

Response: Thank you for pointing this out. The entire manuscript has been thoroughly proofread and edited. Numerous grammatical errors, awkward phrasing, and inconsistencies in writing style have been corrected. All revisions have now been incorporated into the text, thereby enhancing the article’s overall readability and academic quality.

Comment 2: The chemical elements and mechanical properties for the material 6061 aluminum alloy should be added.

Response: A new table (Table 2) has been added in Section 3.2, listing the typical chemical composition (Si, Fe, Cu, Mn, Mg, Cr, Zn, Ti, Al) and key mechanical properties (tensile strength, yield strength, elongation, hardness) of 6061 aluminum alloy.

Comment 3: The information for the finite element model is missing, like type of element? Number of elements and nodes?

Response: We have substantially expanded the description of the finite-element model in Sections 3.1 and 3.2.

Comment 4: What is the unit in Eq(5) for parameter T?

Response: We apologize for the omission. In the revised manuscript, the temperature 𝑇 in the thermal-diffusion equation (now Eq. 2) is explicitly stated to be in Kelvin (K). All units throughout the manuscript have been double-checked to avoid ambiguity.

Comment 5: In Fig. 4, there is Chinese character.

Response: Figure 4 has been updated – all Chinese labels have been replaced with their English equivalents.

Comment 6: “Gaussian and super-Gaussian beams were used as the laser heat sources.” Are there any other types of beams suitable?

Response: Two representative pulse shapes, Gaussian and super-Gaussian, were selected for this study because they exemplify the typical bell-shaped and flat‑top energy distributions, respectively. Other pulse shapes (e.g., triangular or arbitrary waveforms) are beyond the scope of the present work.

Comment 7: “The pulsed laser was assumed to be normally incident at the center of the aluminum-alloy surface.” Is there any reference?

Response: A justification has been added, citing standard modelling practice. The text now reads: “Following common practice in laser-ablation modeling [15], the pulsed laser is assumed to be normally incident at the center of the aluminum-alloy surface.” Reference [15] has been verified to support this assumption.

Comment 8: The discussion is not enough, you should explain more useful information.

Response: The Discussion (Section 5) has been completely rewritten and extended. It now provides: A quantitative analysis of plasma shielding and energy‑coupling efficiency, estimating that up to 30% of laser energy can be shielded by the plasma for Gaussian pulses, compared to 15–20% for super-Gaussian pulses.An interpretation of how the temperature distribution induced by different pulse shapes influences melt‑pool lifetime and recast‑layer formation.Practical guidelines for industrial laser processing: super-Gaussian pulses are recommended for high‑aspect‑ratio drilling due to lower plasma losses, whereas Gaussian pulses may be advantageous for surface texturing where sharp thermal gradients are desired.A discussion of the limitations of the present model and an outlook for future work including fluid dynamics and experimental microstructure characterization.

Reviewer #2

Comment 1: The second chapter, the theoretical model section, provides insufficient details on the construction of the finite element model. For example: The boundary conditions (such as thermal boundary, convection, radiation, etc.) have not been clearly defined; the method of grid division and convergence analysis have not been explained; the type of solver, time step, and nonlinear processing method have not been provided.

Response: We fully agree with this constructive remark. All of the requested details have now been incorporated into Sections 2 and 3:Boundary conditions are explicitly listed (see response to Reviewer #1, Comment 3).Meshing and convergence analysis are described in Section 3.1, including the element type, element count, refinement strategy, and convergence test results. Solver settings are now provided: time‑dependent study with adaptive time stepping, on the residual; phase‑change handled by the enthalpy method; material removal implemented by deactivating elements whose temperature exceeds the boiling point

Comment 2: The content of Part 5, "Discussion", is overly repetitive with the results section and lacks in-depth analysis of the underlying mechanisms.Further exploration is needed on the quantitative relationship between plasma shielding and energy coupling, the influence of different pulse shapes on the molten pool flow and recrystallization structure, as well as the gap and promotion path between these findings and actual industrial applications.

Response: We appreciate this insightful suggestion. The Discussion section has been thoroughly restructured to focus on mechanistic insight rather than summarizing results. Plasma shielding—quantitative relationship of energy coupling: Based on simulation-derived curves of electron density versus time, we estimated the proportion of laser energy absorbed by the plasma using the effective absorption coefficient. This analysis directly explains the 3–18% improvement in energy utilization efficiency observed for ultra-high-Gauss pulses. Although the current model does not account for the fluid dynamics of melt flow, we inferred potential microstructural effects using computationally derived temperature gradients and melt pool lifetime. Literature data on similar alloys were cited to support these qualitative correlations. A new section has been added to bridge the gap between laboratory research and actual manufacturing processes. It explores how optimizing pulse shape can reduce the scrap rate in laser drilling of aerospace components and emphasizes the necessity of online monitoring of plasma emission.

Comment 3: The conclusion section lacks quantitative comparison.Although it is mentioned that "ultra-Gaussian pulses increase energy utilization by approximately 3–18%", no comparison with the literature or description of the applicable range is provided. Suggestion: Add quantitative comparisons of different pulse shapes from existing studies to enhance the persuasiveness of the conclusion.

Response: The Conclusions section has been reinforced by incorporating quantitative comparisons from the recent literature. For instance, studies on beam shaping in metal ablation (e.g., Bakhtari et al., 2024) report efficiency enhancements of 5–20%, which aligns well with our 3–18% range. The applicable parameter window (energy 50–150 mJ, spot radius 1–2 mm) is now explicitly stated, and it is noted that the advantage of super‑Gaussian pulses is most pronounced near the ablation threshold where plasma shielding is relatively weak.

We believe that these revisions fully address all concerns raised by the reviewers. The manuscript has been significantly improved in both depth and clarity. We hope it is now acceptable for publication in PLOS ONE.

Sincerely,

The authors

Attachment Submitted filename: renamed_085e6.docx

# Peer review: Decision Letter 1

**DOI:** 10.1371/journal.pone.0357262.r003

**Authors:** Khalil Abdelrazek Khalil

17 Aug 2026

High-power pulsed-laser ablation of aluminum alloy: simulation model and experimental validation

PONE-D-26-08729R1

Dear Dr. hou,

We’re pleased to inform you that your manuscript has been judged scientifically suitable for publication and will be formally accepted for publication once it meets all outstanding technical requirements.

Within one week, you’ll receive an e-mail detailing the required amendments. When these have been addressed, you’ll receive a formal acceptance letter and your manuscript will be scheduled for publication.

An invoice will be generated when your article is formally accepted. Please note, if your institution has a publishing partnership with PLOS and your article meets the relevant criteria, all or part of your publication costs will be covered. Please make sure your user information is up-to-date by logging into Editorial Manager at Editorial Manager® and clicking the ‘Update My Information' link at the top of the page. For questions related to billing, please contact billing support.

If your institution or institutions have a press office, please notify them about your upcoming paper to help maximize its impact. If they’ll be preparing press materials, please inform our press team as soon as possible -- no later than 48 hours after receiving the formal acceptance. Your manuscript will remain under strict press embargo until 2 pm Eastern Time on the date of publication. For more information, please contact onepress@plos.org.

Kind regards,

Khalil Abdelrazek Khalil, Ph.D.

Academic Editor

PLOS One

Additional Editor Comments (optional):

Reviewers' comments:

Reviewer's Responses to Questions

**Comments to the Author**

Reviewer #1: All comments have been addressed

Reviewer #2: All comments have been addressed

**********

2. Is the manuscript technically sound, and do the data support the conclusions?

Reviewer #1: Yes

Reviewer #2: Yes

**********

3. Has the statistical analysis been performed appropriately and rigorously?

Reviewer #1: Yes

Reviewer #2: Yes

**********

4. Have the authors made all data underlying the findings in their manuscript fully available?

The PLOS Data policy

Reviewer #1: Yes

Reviewer #2: Yes

**********

5. Is the manuscript presented in an intelligible fashion and written in standard English?

Reviewer #1: Yes

Reviewer #2: Yes

**********

Reviewer #1: It is well modified, and can be accepted for accepted for publication. It offers an high-power pulsed-laser ablation of aluminum alloy:simulation model and experimental validation method.

Reviewer #2: It is well modified, and can be accepted for publication. All questions are well addressed. English writing is good.

**********

what does this mean?). If published, this will include your full peer review and any attached files.

If you choose “no”, your identity will remain anonymous but your review may still be made public.

**Do you want your identity to be public for this peer review?**  For information about this choice, including consent withdrawal, please see our Privacy Policy

Reviewer #1: No

Reviewer #2: No

**********

# Editor report: Acceptance letter

**DOI:** 10.1371/journal.pone.0357262.r004

**Authors:** Khalil Abdelrazek Khalil

PONE-D-26-08729R1

PLOS One

Dear Dr. hou,

I'm pleased to inform you that your manuscript has been deemed suitable for publication in PLOS One. Congratulations! Your manuscript is now being handed over to our production team.

At this stage, our production department will prepare your paper for publication. This includes ensuring the following:

* All references, tables, and figures are properly cited

* All relevant supporting information is included in the manuscript submission,

* There are no issues that prevent the paper from being properly typeset

You will receive further instructions from the production team, including instructions on how to review your proof when it is ready. Please keep in mind that we are working through a large volume of accepted articles, so please give us a few days to review your paper and let you know the next and final steps.

Lastly, if your institution or institutions have a press office, please let them know about your upcoming paper now to help maximize its impact. If they'll be preparing press materials, please inform our press team within the next 48 hours. Your manuscript will remain under strict press embargo until 2 pm Eastern Time on the date of publication. For more information, please contact onepress@plos.org.

You will receive an invoice from PLOS for your publication fee after your manuscript has reached the completed accept phase. If you receive an email requesting payment before acceptance or for any other service, this may be a phishing scheme. Learn how to identify phishing emails and protect your accounts at https://explore.plos.org/phishing.

If we can help with anything else, please email us at customercare@plos.org.

Thank you for submitting your work to PLOS One and supporting open access.

Kind regards,

PLOS One Editorial Office Staff

on behalf of

Dr. Khalil Abdelrazek Khalil

Academic Editor

PLOS One
